# Advances in Printed Electronic Textiles

**DOI:** 10.1002/advs.202304140

**Published:** 2023-11-27

**Authors:** Md Rashedul Islam, Shaila Afroj, Junyi Yin, Kostya S. Novoselov, Jun Chen, Nazmul Karim

**Affiliations:** ^1^ Centre for Print Research (CFPR) University of the West of England Frenchay Campus Bristol BS16 1QY UK; ^2^ Department of Bioengineering University of California Los Angeles CA 90095 USA; ^3^ Institute for Functional Intelligent Materials Department of Materials Science and Engineering National University of Singapore Singapore 117575 Singapore; ^4^ Nottingham School of Art and Design Nottingham Trent University Shakespeare Street Nottingham NG1 4GG UK

**Keywords:** E‐textiles, printing, wearables, personalized healthcare

## Abstract

Electronic textiles (e‐textiles) have emerged as a revolutionary solution for personalized healthcare, enabling the continuous collection and communication of diverse physiological parameters when seamlessly integrated with the human body. Among various methods employed to create wearable e‐textiles, printing offers unparalleled flexibility and comfort, seamlessly integrating wearables into garments. This has spurred growing research interest in printed e‐textiles, due to their vast design versatility, material options, fabrication techniques, and wide‐ranging applications. Here, a comprehensive overview of the crucial considerations in fabricating printed e‐textiles is provided, encompassing the selection of conductive materials and substrates, as well as the essential pre‐ and post‐treatments involved. Furthermore, the diverse printing techniques and the specific requirements are discussed, highlighting the advantages and limitations of each method. Additionally, the multitude of wearable applications made possible by printed e‐textiles is explored, such as their integration as various sensors, supercapacitors, and heated garments. Finally, a forward‐looking perspective is provided, discussing future prospects and emerging trends in the realm of printed wearable e‐textiles. As advancements in materials science, printing technologies, and design innovation continue to unfold, the transformative potential of printed e‐textiles in healthcare and beyond is poised to revolutionize the way wearable technology interacts and benefits.

## Graphical abstract

This work covers key aspects of fabricating printed wearable e‐textiles, including material selection, printing techniques, and their potential applications. As advancements in materials science, printing technologies, and design innovation continue to unfold, the transformative potential of printed e‐textiles in healthcare and beyond is poised to revolutionize the way humans interact with and benefit from wearable technology.

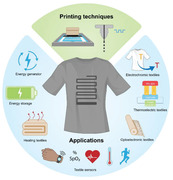

## 1 Introduction

Fusing electronics into daily textiles, i.e., e‐textiles, being sufficiently soft to wear and smart to interact with their surroundings, holds great potential to transform the current healthcare that based on disease management into a personalized model based on disease prevention and healthcare enhancement.^[^ ^1^, ^2^, ^3^ ^]^ By definition, e‐textiles refer to electronic devices, that can be attached to the body or clothes^[^ ^4^, ^5^ ^]^ comfortably and un‐obstructively to deliver intelligent assistance.^[^ ^6^ ^]^ With the rapid advancement of the Internet of Things, intelligent hardware, and big data, e‐textiles have been growing in momentum since the 2000s,^[^ ^7^ ^]^ with great potential in health care, energy, education, social networking, the military, etc.^[^ ^8^, ^9^ ^]^ Advantages such as great flexibility, light weight, breathability, and ease of integration with traditional clothes, make e‐textiles an ideal candidate platform as wearable bioelectronics.^[^ ^10^, ^11^, ^12^ ^]^ Such electronics can be worn either internally as implantable devices or externally as skin‐interfaced devices.^[^ ^8^, ^13^ ^]^ Many of these technologies have become popular parts of people's daily lives as lifestyle and healthcare products, including fitness tracking, rehabilitation, fall detection, and even wound healing.^[^ ^14^, ^15^ ^]^ The regular and continuous real‐time monitoring of various human health indicators, i.e., heart rate, blood pressure, glucose level, body temperature, etc. noninvasively can alert users and health care providers about abnormal and/or urgent critical indications.^[^ ^16^, ^17^, ^18^ ^]^ It enables cost‐effective and affordable personalized healthcare at any time and place and obviates the need for advanced healthcare facilities and skilled healthcare professionals.^[^ ^19^ ^]^ Additionally, such soft bioelectronics can detect various signals with high sensitivity, with the potential to be used in artificial electronic skin, motion detection, telemedicine, and in‐home healthcare.^[^ ^20^, ^21^ ^]^ Considerable efforts have been devoted over the past few years to the development of wearable electronics through advancements in new materials, new processes, and sensing mechanisms.^[^ ^20^ ^]^

**Figure 1:**
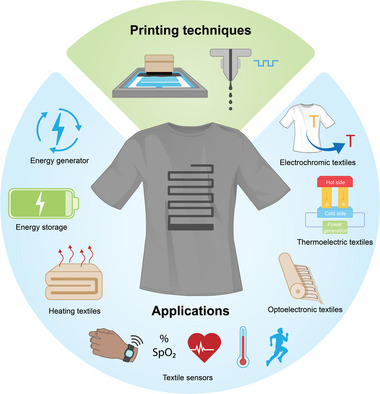
Printing techniques and printed electronic textiles (e‐textiles) for energy and healthcare.

The development of electronic textiles essentially requires the fabrication of electro‐active materials onto textiles. 1D‐shaped conductive textiles are generally prepared by spinning inherently conductive polymer or mixing electroactive material with the spinning dope and/or surface coating of fiber/filament. Padding and/or coating methods are generally used when conductive 2D plane‐shaped fabric/textiles are required. A thorough deposition of electroactive material occurs in such cases. All these processes require the utilization and disposal of a huge quantity of conductive materials that are often toxic, environmentally harmful, and non‐biodegradable. Printing, i.e., localized deposition of active material in the form of printing ink/paste onto textiles, could be a sustainable solution as the material disposal is significantly low compared to yarn/fabric coating process. Additionally, the printing technique offers simple, low‐cost, timesaving, versatile, and environmentally friendly manufacturing technologies on various textile substrates. Therefore, the next trend for wearable e‐textile fabrication is the printing of all electronic parts onto textiles,^[^ ^22^ ^]^ (**Figure** **1**).

**Figure 2:**
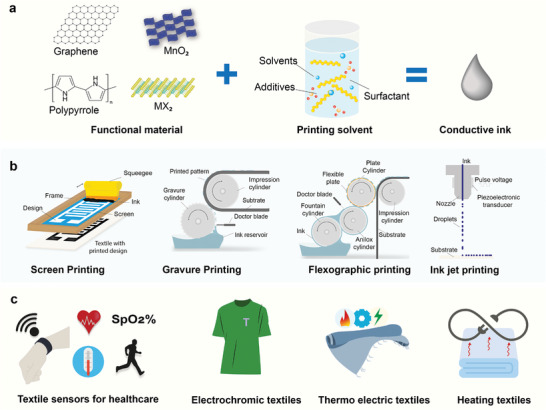
Print ink and printing processes for e‐textiles a) Preparation of conductive inks b) Printing techniques for e‐textiles fabrication and c) Applications of printed e‐textiles.

There is no doubt that next‐generation printed flexible and wearable electronics will lead to a revolution in the human way of life.^[^ ^20^ ^]^ Though great progress has been made in the field of e‐textiles, most of the reported results are still far from commercial adoption for practical applications. The obstacles mainly come from the aspects namely materials, manufacturing techniques, integration, testing standards, and methods for wearable technology. Currently, there is a lack of comprehensive review on the material, manufacturing, and performance of the printed flexible wearable e‐textiles, which is exactly the purpose of this review. In this review (**Figure** **2**), first, we introduced the scope of our review in Section 1. Additionally, conductive materials and suitable textiles for the fabrication of e‐textiles are summarized in Section 2. Section 3 introduces the printing techniques and the requirements for various functional printing techniques are discussed in Section 4. Applications of printed e‐textiles in terms of various wearable sensors, energy storage devices, and heating textiles are reviewed in Section 5. We finally conclude our review presenting our outlook on various aspects of printed wearables.

## 2 Materials for Printed E‐textiles

### 2.1 Functional Materials for Printed E‐textiles

**Figure 3:**
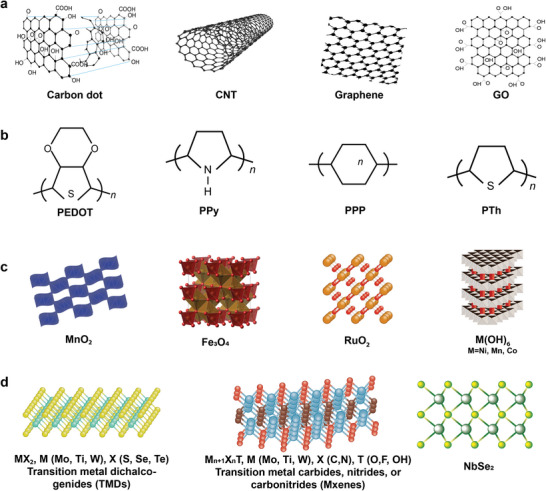
Printable functional materials for e‐textiles a) Carbonaceous materials b) Conductive polymers c) Metal oxides/hydroxides and d) 2D materials.

Development of smart/intelligent and interactive printed e‐textiles requires fabrication of flexible and durable conductive components which is an integrated part of the textile. Therefore, the first and an important element in fabricating e‐textiles is developing the electrically conductive printing ink/paste,^[^ ^23^ ^]^ to ensure the flow of electrons, i.e., electrical current within the printed pattern. SI unit of siemens per meter (S m^−1^) is commonly used to measure electrical conductivity (σ).^[^ ^24^ ^]^ Alternatively, resistivity (inversely proportional to conductivity) is used to measure the electrical properties of materials. Sheet resistance is used for 2D materials with a consistent thickness (ohms per square meter, Ω m^−2^) and linear resistance is measured for 1D conductive threads (ohms per unit length, Ω m^−1^). Additionally, other characteristics, including thermal and mechanical properties, chemical and thermal resistance, weight and density, heat removal efficiency, interconnections with traditional wires, reliability, and durability, are also considered for textile‐based conductor preparation.^[^ ^25^ ^]^ **Figure** **3** summarizes the commonly used functional materials for the fabrication of e‐textiles.

#### 2.1.1 Carbonaceous Materials

Carbonaceous compounds and their allotropes (Figure 3) are key materials for applications in nano and optoelectronics, photonics, energy generation and storage, nano‐mechanics, and catalysis, and are therefore, topics of particular interest for the researchers.^[^ ^26^ ^]^ In addition to high electrical conductivity, they possess a unique combination of other chemical and physical properties, including exceptionally high Young's modulus, mechanical strength, light transmittance, surface‐area range (≈1 to 2000 m^2^ g^−1^), temperature stability, good corrosion resistance, controlled pore structure, processability and compatibility with various materials, and are relatively cheap.^[^ ^27^, ^28^ ^]^

Carbon nanotube (CNT) is a 1D allotrope of carbon. They are nano‐meter in diameter and several millimeters in length and are available in the form of single‐walled carbon nanotubes, SWCNTs, or multiwalled carbon nanotubes, MWCNTs (by rolling up single or multiple sheets of graphene respectively).^[^ ^29^ ^]^ Since its discovery in the 1990s, CNTs have been utilized in a variety of applications including actuators, artificial muscles, and lightweight electromagnetic shields.^[^ ^30^ ^]^ Their exceptional physical, chemical, and electronic properties offer exciting possibilities for nano‐ scale electronic applications.^[^ ^31^ ^]^

Carbon black (CB), has become an interesting material for sensor fabrication in recent years, due to its excellent conductive and electrocatalytic properties, as well as its cost‐effectiveness.^[^ ^32^ ^]^ Conductive CBs usually possess electrical conductivity in the range of 10^−1^ to 10^2^ (Ω cm)^−1^ with high porosity, small particle size, and a chemically clean (oxygen‐free) surface.^[^ ^27^ ^]^ CBs are manufactured generally by thermal decomposition, including detonation, or by incomplete combustion of carbon‐hydrogen compounds.^[^ ^33^ ^]^

Activated carbon (AC) powder, in comparison with the other forms of carbonaceous materials, is known as a good electrode material with a specific surface area of 1000–2000 m^2^ g^−1^,^[^ ^34^ ^]^ inexpensive and environmentally friendly alternative.^[^ ^35^ ^]^ The tuneable pore size, high specific surface, hierarchical pore structure, and different morphology enable the formation of bilayer of ions at electrode‐electrolyte interfaces of AC,^[^ ^36^ ^]^ which is beneficial for energy storage applications.

Graphene is a 2D allotrope of carbon, which is the basic structural element of carbon allotropes including graphite, carbon nanotubes, and fullerenes.^[^ ^37^ ^]^ Since its isolation in 2004, graphene received much attention from the research community due to its outstanding mechanical, thermal, electrical, and other properties which unveiled a wide range of other similar 2D materials.^[^ ^38^, ^39^, ^40^, ^41^ ^]^ It is considered as the “mother” of all graphitic‐based nanostructures, due to the variety of sizes and morphologies. In addition to a single‐layer structure, graphene can also be stacked into multi‐layered sheets.^[^ ^42^ ^]^ Mechanical, thermal, and liquid phase exfoliation and chemical vapor deposition (CVD) are the common techniques to manufacture graphene.^[^ ^43^, ^44^ ^]^ The unique physicochemical properties including theoretical high specific surface area (2600 m^2^ g^−1^), good biocompatibility, strong mechanical strength (130 GPa), excellent thermal conductivity (3000 Wm^−1^ K^−1^), high electrical charges mobility (230 000 cm^2^ V^−1^ s^−1^) and fast electron transportation makes it not only a unique but also a promising material for next‐generation electronics applications.^[^ ^45^, ^46^, ^47^ ^]^ Graphene and its derivatives have the capability to form chemical bonds with textiles and therefore, offer great potential to be used in wearable smart e‐textiles.^[^ ^48^, ^49^, ^50^, ^51^ ^]^ Graphene oxide (GO),^[^ ^52^ ^]^ possesses strong mechanical, electronic, and optical properties, chemical functionalization capability, large surface area, high stability, and layered structure.^[^ ^53^, ^54^, ^55^ ^]^ Based on the degree of oxidation, it acts as a semiconductor or insulator, enabling usage in many fields.^[^ ^56^ ^]^ It is obtained by treating graphite materials with strong oxidizing agents which loosen the tightly stacked graphite layers by introducing oxygen atoms to the carbon,^[^ ^57^ ^]^ and forming single‐layer graphite oxide sheets.^[^ ^58^ ^]^ Reduced graphene oxide (rGO), is another important derivative of graphene exhibiting properties between graphene and GO.^[^ ^59^, ^60^ ^]^ It consists of few‐atom‐thick 2D sp^2^ hybridized carbon layers with fewer oxygeneous compounds. Though resembles graphene, residual oxygen, and other heteroatoms with possible structural defects degrade its electric properties.^[^ ^61^ ^]^ Graphene derivatives (GO and rGO) can be produced in large quantities in their stable dispersions,^[^ ^62^ ^]^ however, the major challenge is the production of high‐quality graphene at a larger scale.^[^ ^63^ ^]^ Hybridization of various carbonaceous compounds is also attractive in wearable electronics applications.^[^ ^64^, ^65^ ^]^

#### 2.1.2 Conductive Polymers

Conductive polymers (CPs) are organic polymers that conduct electricity through a conjugated bond system along their polymer chain. Over the past two decades, CPs have been widely studied as a promising active material for wearable electronics owing to their reversible faradaic redox nature, high charge density, and low cost in comparison to metal oxides.^[^ ^66^ ^]^ The era of intrinsic CPs started with the invention of polyacetylene; however, Polyaniline (PANI) attracted much more attention due to its cheaper monomer compared to polyacetylene and ease of synthesis.^[^ ^67^ ^]^ PANI offers multi‐redox reactions, high conductivity, high specific capacitance, and excellent flexibility,^[^ ^68^ ^]^ thus playing a great role in flexible electronics, energy storage, and conversion devices. Sometimes, the use of PANI is recommended with other active materials (carbons, metals, or other polymers) due to its instability issue.^[^ ^69^, ^70^ ^]^

Polypyrrole (PPy) is a π‐electron conjugated CP that offers large theoretical capacitance, good redox properties, superior conductivity, ease of synthesis, nontoxicity, biocompatibility, and high thermal and environmental stability.^[^ ^71^ ^]^ Similar to PANI, PPy also possesses intrinsic disadvantages, such as brittleness. The processability and mechanical properties can be improved by blending PPy with other fiber polymers or forming copolymers of PPy.^[^ ^72^ ^]^ This PPy‐based composite material can demonstrate electrical properties similar to metals or semiconductors while used for wearable applications.^[^ ^73^ ^]^ Good water solubility and safety (less carcinogenic than PANI), make PPy a suitable electrode material for wearable electronics.^[^ ^74^, ^75^ ^]^

Poly(3,4‐ethylenedioxythiophene), PEDOT is another π‐conjugated polymer that exhibits properties such as excellent conductivity (≥300 S cm^−1^), electro‐optic properties, and processability.^[^ ^76^, ^77^ ^]^ Though PEDOT is usually nonconductive or shows very little conductivity in its undoped state, it is highly conductive in its oxidized (doped) state. However, the low stability limits its industrial application. Several approaches have been considered to tackle this issue including: the addition of conducting nanofillers to increase conductivity and mixing or depositing metal oxide to enhance capacitance.^[^ ^78^ ^]^ For example, the polymer combination with polystyrene sulfonate, i.e., PEDOT:PSS, possesses a high conductivity (up to 4600 S cm^−1^),^[^ ^79^ ^]^ and thus offers promise as an electrode material for wearable electronics.^[^ ^80^, ^81^ ^]^ It is known to be a mixed conductor to function as both an ionic and electronic conductor, where PEDOT is responsible for electronic conductivity, and PSS contributes to ionic conductivity.^[^ ^82^ ^]^ It is also considered as environmentally stable, and decently biocompatible.^[^ ^83^ ^]^ The hybridization of PEDOT:PSS with other active materials (such as carbonaceous compounds) has also been studied for electrode fabrication.^[^ ^84^, ^85^ ^]^

#### 2.1.3 Metals and their Oxides

Production of conductive components is currently based on carbon nanotubes, graphene, organometallic compounds, conductive polymers, and metal nanoparticles. However, the high conductivity values make metal nanoparticles as one of the most effective components for producing conductive tracks. They possess conductivity 2–4 orders of magnitude higher (10^4^–10^5^ S cm^−1^) than the CPs, carbon, and graphene, (10–10^2^ S cm^−1^).^[^ ^86^ ^]^ Noble metal particles such as gold (Au), silver (Ag), and copper (Cu) are mostly exploited as potential conductive materials in printed electronics.^[^ ^87^ ^]^

Silver (Ag) has the highest electrical conductivity (6.3 × 10^7^ S m^−1^) among the metals, which facilitates direct electronic conduction.^[^ ^88^ ^]^ Thus Ag nanoparticles are considered as a suitable choice for the formulation of conductive inks. In addition to high conductivity, it has other advantages including low melting point, high resistance to oxidation, and a feasible processing method.^[^ ^86^ ^]^ Therefore, silver is the material of choice for printed electronics^[^ ^22^, ^89^ ^]^ and has been used in various substrates via various fabrication techniques; spin coating on PI,^[^ ^90^ ^]^ inkjet^[^ ^91^ ^]^ or nozzle‐jet^[^ ^92^ ^]^ printing on PET, inkjet printing on PI,^[^ ^93^ ^]^ etc. However, the high cost of Ag limits its commercial use. Copper (Cu), as an alternate material to Ag, has been actively studied due to its lower cost (about only 1% of Ag) and high electrical conductivity (about only 6% less than that of Ag).^[^ ^94^ ^]^ However, Cu easily gets oxidized during the synthesis process and post‐treatments such as washing, mixing, and storage in ambient conditions.^[^ ^95^ ^]^ Due to high intrinsic conductivity (2.44 µΩ·cm), Gold (Au) is considered one of the highly conductive metal inks for wearable electronics.^[^ ^96^ ^]^ It also offers good ductility, reliable performance, and easy fabrication.^[^ ^97^ ^]^

Metal oxides, on the other hand, due to their wide variety of oxidation states, are generally considered as prime candidates to use as electrode material in energy storage devices.^[^ ^98^ ^]^ Metal oxide electrodes possess exceptional properties, which makes them suitable for a wide range of applications including sensors, semiconductors, energy storage, lithium‐ion batteries, solar cells, etc.^[^ ^99^ ^]^ Ruthenium dioxide (RuO_2_) possesses a high theoretical specific capacitance value (1400–2000 F g^−1^),^[^ ^100^ ^]^ demonstrates highly reversible redox reactions, good thermal stability, high electronic conductivity (300 S cm^−1^), and superior cycle lifespan with high rate capability.^[^ ^98^ ^]^ All these properties make RuO_2_ as a promising material for supercapacitor (SC) devices.^[^ ^101^, ^102^ ^]^ Manganese dioxide (MnO_2_) is considered another promising electrode material for electrochemical capacitors,^[^ ^103^ ^]^ due to its low cost, high theoretical specific capacitance (≈1370 F g^−1^), natural abundance, nontoxicity, and environmental friendliness.^[^ ^104^ ^]^ Nickel oxide (NiO, is considered another attractive conversion reaction‐based anode material in the field of supercapacitors due to its low cost, ease of preparation, environment friendliness, nontoxicity, and high theoretical capacity (≈3750 F g^−1^).^[^ ^105^ ^]^ Additionally, Nickel hydroxide [Ni(OH)_2_] is another attractive electrode material for supercapacitors due to its high theoretical capacity and superior redox behavior.^[^ ^106^, ^107^ ^]^ Furthermore, Cobalt oxide (Co_3_O_4_) also possesses superior reversible redox behavior, excellent cycle stability, large surface area, and outstanding corrosion stability,^[^ ^108^, ^109^, ^110^ ^]^ thus another suitable electrode material for supercapacitor electrodes. Due to its layered structure with a large interlayer spacing, Cobalt hydroxide [Co(OH)_2_] provides a large surface area with a high ion insertion/extraction rate offering a great potential to become a high‐performance electrode material^[^ ^98^ ^]^ specifically for energy storage studies.^[^ ^110^, ^111^ ^]^ Fe_3_O_4_, TiO_2_, SnO_2_, V_2_O_5,_ etc are also other metal oxides used for electrode fabrication. Despite having such outstanding properties, a few problems still exist that hinder their practical application. Such as the production cost of RuO_2_ is higher, and it also suffers from the agglomeration effects.^[^ ^100^ ^]^ MnO_2_ possesses poor conductivity, considerably lower actual specific capacitance than the theoretical, poor structural stability, and easy dissolving nature in the electrolyte resulting in poor cycling ability._,_^[^ ^111^ ^]^ NiO also has relatively poor electrical conductivity and lower specific surface area. Similarly, Ni(OH)_2_ suffers from lower conductivity, and poor stability, with large volume changes during charge–discharge processes. As a consequence, the combination of metal oxides with other active components is much preferred by the researchers for electrode application.^[^ ^112^, ^113^, ^114^, ^115^, ^116^ ^]^

#### 2.1.4 2D Materials

The discovery of graphene has unveiled a wide range of graphene‐like 2D materials (2DM) with outstanding properties.^[^ ^38^ ^]^ 2DMs such as transition metal chalcogenides (TMDs)‐ Molybdenum disulfide (MoS_2_), Tungsten selenide (WSe_2_), hexagonal boron nitride (h‐BN), transition metal carbides/nitrides (i.e., MXenes‐ Ti_2_C) and 2D metal‐organic frameworks (MOFs) attracted tremendous research attention due to their extraordinary properties such as large surface area, good electronic conductivity, excellent electrochemical properties, and good chemical, electrochemical, and thermal stability.^[^ ^117^, ^118^, ^119^ ^]^ 2DMs are generally defined as materials with infinite crystalline extensions along two dimensions and one crystalline dimension with few or single atomic layers thickness.^[^ ^120^ ^]^ However poor cyclic stability, and large structural changes during metal‐ion insertion/extraction, as well as higher manufacturing cost are the major challenges for 2DMs which require further improvements to find their wide applications in wearable electronics.^[^ ^121^ ^]^

MoS_2_, an exciting 2D material due to its graphene‐like properties has been investigated to a lesser extent but is gaining increased interest nowadays for integration into electronic devices.^[^ ^122^ ^]^ In addition to conventional synthesizing processes such as micromechanical peeling or chemical vapor deposition, MoS_2_ is currently being synthesized by ultrasonic treatment similar to graphene. Large volumes of monolayer and few‐layer flakes can be produced which can further be deposited onto a substrate or formed into films.^[^ ^123^ ^]^ Their favorable electrochemical properties are mainly a result of the hydrophilicity and high electrical conductivity, as well as the ability of the exfoliated layers to dynamically expand and intercalate various ions.^[^ ^124^ ^]^ Hexagonal boron nitride (2D‐hBN), an isomorph of graphene,^[^ ^125^ ^]^ is uniquely featured by its exotic optoelectrical properties together with mechanical robustness, thermal stability, and chemical inertness. Though an insulator itself, strategies such as doping, substitution, functionalization, and hybridization can tune its properties and functionalities, making it a truly versatile functional material for a wide range of applications. BN‐based nanomaterials alone or in combination with other 2DMs, have huge potential in next‐generation microelectronic and other technologies,^[^ ^126^ ^]^ especially electrodes, electrochemical energy storage and conversion.^[^ ^127^ ^]^

MXene is a relatively new family of 2D metal carbides, nitrides, and carbonitrides, with unique intrinsic physical/chemical properties, that have thoroughly been investigated and can be used in various research fields, including ceramics, energy storage, sensors, water purification, catalysis, thermoelectric and photothermal conversion, solar cell, biomedicine, and microwave absorption and shielding.^[^ ^128^, ^129^ ^]^ 2D MXenes possess attractive electrical and electrochemical properties including hydrophilicity, conductivity, surface area, topological structure, rich surface chemistry, tunable terminations, excellent processability, etc.,^[^ ^130^, ^131^, ^132^ ^]^ and thus gained much attention in electrochemistry. The term MXenes (with a formula of M_n+1_X_n_) are named after other 2D analog materials silicene, graphene, phosphorene, and so on, synthesized by extracting an atomic layer from ternary MAX (M_n+1_AX_n_) ceramics, where M = early transition metal elements (Ti, Zr, Mo, Nb, V, Mn, Sc, Hf, W, and so on), A = group 13 or 14 (Si, Al, Ga, and so on), X = C or/and N. In addition to being used as alone, the improved coupling and hybridization of MXene with other materials at the nano‐scale makes it one of the most intriguing materials for wearable applications.^[^ ^133^ ^]^

### 2.2 Textile Substrates for Printed E‐textiles

With the development of technology and a variety of requirements, the demand for smart materials and intelligent textiles growing rapidly all over the world. A multifunctional wearable electronic device requires a conformal platform close to the human body, thus textile or fabric that is usually embedded with normal clothes and worn on various body parts has emerged as a promising substrate and platform for wearable electronics. Textiles with basic characteristics of being soft, flexible, air‐permeable, low‐cost, and integrable with various forms of garments^[^ ^134^, ^135^ ^]^ are commonly made of natural cotton, silk, or wool fibers or synthetic poly (ethylene terephthalate) (PET) or polyester, polyamide or nylon, polypropylene, viscose rayon filament.^[^ ^136^ ^]^ Additionally, there are polyimide (PI), thermoplastic, polyethylene naphthalate (PEN), and thermoplastic polyurethane (TPU) based textiles in use. These textiles vary in their physical, chemical, thermal, and tensile properties and therefore, the choice of any specific substrate depends on the properties required for the end‐product. For example, PET excels in applications requiring a smooth surface of a few nanometres of thickness and optical transparency. PI possesses high glass transition temperatures and relatively higher mechanical as well as chemical strength in comparison to PET and is widely preferred for fabricating flexible printed circuit boards (PCBs). TPU and polydimethylsiloxane (PDMS) are suitable for developing stretchable devices, whereas paper is suitable for cheap and disposable devices. Textile‐based substrates offer several advantages over plastic or paper‐based substrates when flexibility and stretchability are concerned. For instance, the porous structure of textiles provides abundant support for the loading of active materials, facilitating rapid absorption due to their hydrophilic nature, resulting in much higher areal mass loading. Therefore, low‐cost, and highly efficient textile‐based wearable electronics have already gained the potential to be used for future high‐tech sportswear, work wear, portable energy systems, military camouflages, and health monitoring systems.^[^ ^137^, ^138^ ^]^

The surface wettability of a polymeric material is of great importance when it comes to different applications in material science.^[^ ^139^ ^]^ This is a basic feature of printing substrate which is a direct intermolecular interaction occurring when liquid and solid medium are brought together. The study of wettability involves the measurements of contact angle (CA), indicating the degree of wetting when a solid and liquid interact. CA when low (<90°) corresponds to high wettability, meaning that the fluid will spread over a large surface area. A high CA (>90°) represents low wettability, so the fluid will minimize contact with the surface and form a compact liquid droplet. CA>150° indicates minimal contact between the liquid droplet and the surface and it corresponds to a superhydrophobic behavior.

Surface tension, or surface free energy, both similar parameters, corresponds to the residual binding capacity of a material surface, i.e., the binding capacity of atoms or groups of atoms that constitute the border surface of the material of interest.^[^ ^140^ ^]^ When talking about solid surfaces only, the term “free surface energy” is usually used. Both terms refer to the same physical quantity and share the same symbol. The unit of the free surface energy is Jm^−2^, and the unit of surface tension is Nm^−1^, which is, once multiplied by m m^−1^ the same unit.^[^ ^141^ ^]^ The values of surface free energy of some non‐modifiable polymer films are in **Table** **1**.

**Table 1:** Specification of various substrates employed for e‐textile fabrication.^[^ ^142^, ^143^ ^]^

Flexible textiles/ polymer substrates	Surface energy (mN m^−1^)	Transparency (%)	Water absorption (%)	Solvent resistance	Folding endurance (cycles)	Dimensional stability	Thickness (µm)	Surface roughness	Density (g/cc)	Glass transition temperature (Tg)	Young modulus (GPa)
PET	44.0	90	0.6	Good	>800	Good	13–356	Poor	1.38	81–150	2–3.2
Polycarbonate (PC)	34.2	92	0.16‐0.35	Poor		Fair		Good		145	2.0–2.6
Polyurethane (PU)	38	‐	0.2	Good	2 000 000	Good	25–500		1.32	80	2.41
Polyimide (PI)	43.8	35‐60	1.3‐3.0	Good	5000‐285 000	Fair	25–125	Good	1.42	360‐410	2.76
PDMS	20.4	‐	>0.1	Poor		Good	125–4775		0.97	145–150	0.57–3.7
Polypropylene (PP)	30.2	84.0‐90.0	0.01	Good		Good		Good		0	0.008–8.25
Polyacrylate (Pacr)	‐	>90	0.2	Good		Good		Fair		105	2.4–3.4
PEN	‐	88	0.3‐0.4	Good	>1000	Good	12.5–250	Good	1.33	120–200	2.2–3
Polyethersulphonate (PES)	‐	89.0	1.4	Poor		Fair		Good		223	2.2
Polycyclic olefin (PCO) /polynorbornene (PNB)	‐	91.6	0.03	Good		Good		Poor		35	1.9

### 2.3 Textile Surface Engineering and Post‐Treatment

The key motivation behind the development of smart textile technologies is providing additional functionalities to common textiles and consequently to a garment.^[^ ^144^ ^]^ Functional textiles can be prepared in two ways. One way is to prepare the functional fiber first and then weave or knit it into a textile. This is achieved by blending and composite spinning of the fiber solution with the functional additives of uniform dispersion to achieve fiber functionalization. The other way is to modify ordinary textiles with functional finishes by coating or printing. The main drawback of this technique is the weak bonding force between the finishing agent and textiles. However, its strength is more durable, and functionality can be maintained for a long time.^[^ ^145^ ^]^

**Figure 4:**
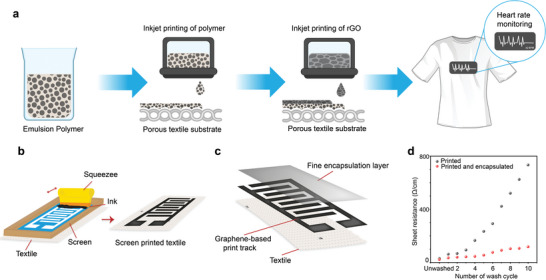
Textile surface pre‐treatment and post‐treatment for e‐textiles. a. Inkjet printing of organic nanoparticle coating followed by inkjet printing of conductive rGO to prepare conductive textile. Reproduced with permission.^[^ ^147^ ^]^ Copyright 2017, The Royal Society of Chemistry. Screen printing of b. graphene ink on textiles c. Polyurethane (PU) based encapsulation post‐treatment to improve washability d. Comparison of the change of electrical resistance of printed textiles with and without encapsulation layer. Reproduced with permission.^[^ ^151^ ^]^ Copyright 2022, The Authors.

Printed electronics often require a uniform and smooth substrate that is also solvent resistant, chemically and thermally stable, stretchable, conformal, flexible, and light weight. One of the key challenges with printed e‐textiles is the ability to achieve continuous highly conductive electrical tracks on a rough and porous textile substrate. Due to the orientation of fibers or yarns, textile fabrics demonstrate an intrinsic planar anisotropy of the general properties.^[^ ^146^ ^]^ Also, the morphology of the fiber changes constantly due to the exchange of water molecules with surroundings, which makes it extremely difficult to produce uniform and continuous conductive paths using low viscous inkjet inks.^[^ ^147^ ^]^ A number of researches have been conducted to introduce an interface layer for reducing such roughness and porosity of textiles. Previous studies have suggested polyurethane acrylate‐based interface layers,^[^ ^148^ ^]^ however the deposition of such interface layers suffers from a few drawbacks. These usually constrain the potential feature resolution, not suitable for small quantities of material deposition or roll‐to‐roll manufacturing. In our previous work,^[^ ^147^ ^]^ we reported an organic nanoparticle‐based inkjet printable textile surface pre‐treatment which enables the production of all inkjet‐printed graphene‐based wearable e‐textiles which is breathable, comfortable and environmentally friendly, **Figure** **4**a.

Another key challenge of the wide commercial adoption of textile‐based wearable electronics is their poor stability on repeated laundry washing.^[^ ^149^ ^]^ Good washability is essential for e‐textiles to survive intense mechanical deformations and water invasion of washing cycles used during their life cycles.^[^ ^150^ ^]^ The delamination of the conductive functional materials due to the mechanical forces experienced during washing cycles, results in a loss the electrical performance.^[^ ^151^ ^]^ The wash stability of wearable e‐textiles can be improved via a number of methods including a textile surface pre‐treatment with Bovine Serum Albumin (BSA), or by a post‐treatment for instance embedding with PDMS, polyurethane (PU) sealing, a screen‐printed PU top layer, and transferred mold or, hot melt encapsulation to seal conductive track on the textile surface.^[^ ^152^ ^]^ In our other work,^[^ ^151^ ^]^ we used a translucent, thin, and stretchable PU‐based encapsulant to protect screen‐printed graphene‐printed wearable e‐textiles, Figure 4b,c. Such encapsulation material adheres with textile materials, keeping the printed graphene layer attached to textiles but covered and protected even during repeated wash cycles, Figure 4d.

Textiles with other functional properties such as antimicrobial, water resistance or water proofing, soil resistance, self‐cleaning, thermoregulations, etc are also of particular interest for various functional applications.^[^ ^153^ ^]^ For example, efforts at the development of functional textiles with antibacterial effects have accelerated recently to provide protection against airborne bio‐particles and micro‐organisms.^[^ ^154^ ^]^ Especially in healthcare environments, textiles with antibacterial properties form a significant part of the hygienic regime of surgical procedures, preventing the spread of infectious pathogens to both patients and staff. Antibacterial finishing thus provides the textiles an improved resilience against microorganisms to prevent the destruction of fibers and discoloration and increases the durability of the textiles with longer life by minimizing microbial colonization of textiles and the potential for transfer from fabric surfaces.^[^ ^155^ ^]^ Numerous methods have been developed in this context to impart antibacterial properties to textiles namely padding, spraying, coating, printing, or foam finishing.^[^ ^156^ ^]^

## 3 Printing Techniques for E‐textiles

The printing process involves a controlled deposition of material, either for decorative or functional purposes, onto a substrate in such a manner that a pre‐defined pattern is produced. Though other deposition processes including painting or spraying are there, printing is further highlighted because it can rapidly produce identical multiples of the original. Three basic methods of printing are there; positive contact, negative contact, and non‐contact printing. Since the substrate is touched by the print master during printing, the first two methods are described as contact printing. Positive contact type resembles the stamping principle, examples include printing presses and woodcuts. Gravure or screen printing are examples of negative contact printing. In the non‐contact printing process, the printer does not come in contact with the substrate. The most common example of non‐contact printing is inkjet printing where ink is ejected on a substrate from a nozzle.^[^ ^157^ ^]^ The available print technologies are briefly introduced in the following section.

### 3.1 Screen Printing

**Figure 5:**
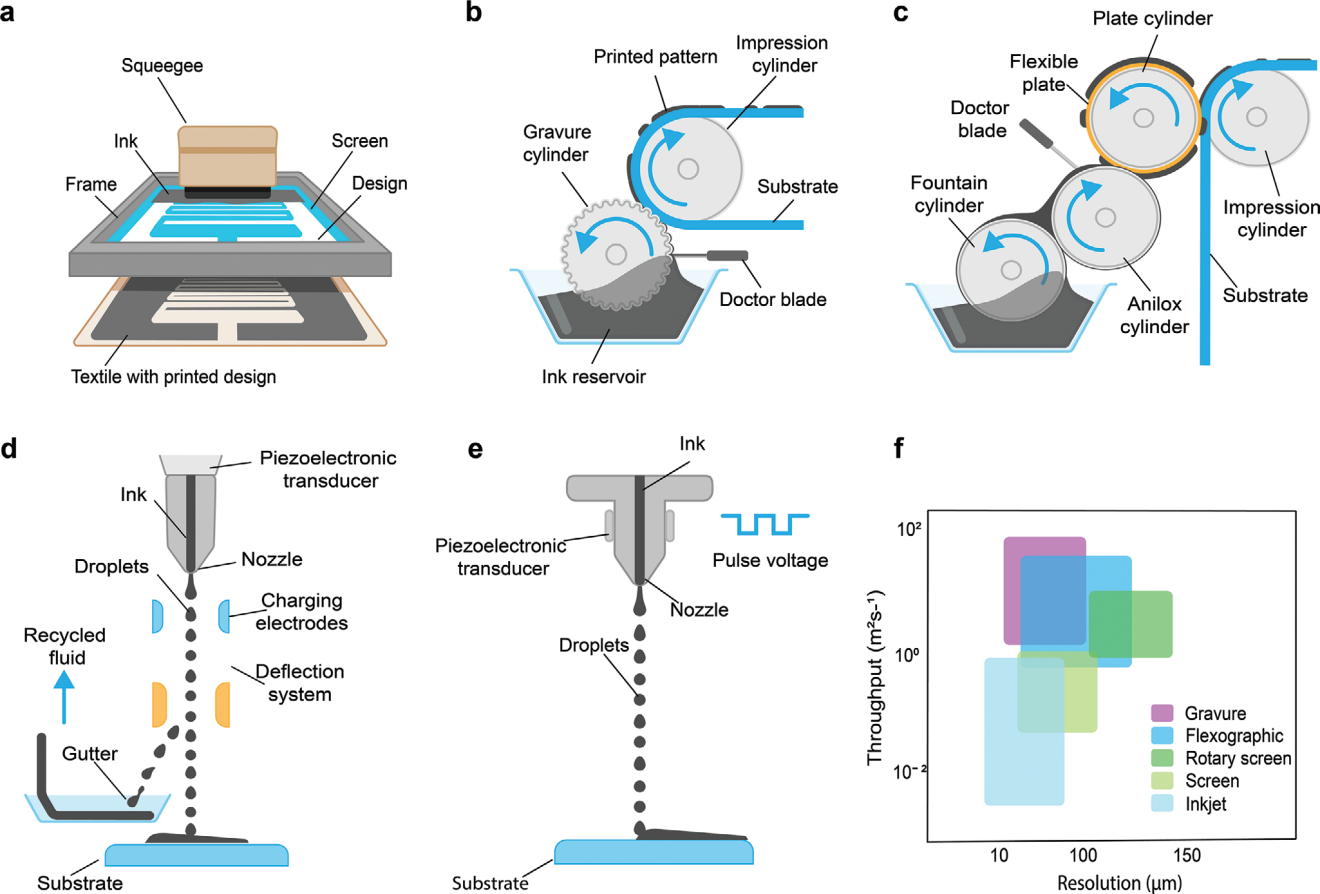
Printing technologies for e‐textiles fabrication, a) Screen printing b) Gravure printing c) Flexographic printing d) Continuous inkjet printing, and e) Drop‐on‐demand inkjet printing f) Comparison of print throughput and best achievable resolution ranges of print technologies. Reproduced with permission.^[^ ^173^ ^]^ Copyright 2018, The Royal Society of Chemistry.

Screen printing (**Figure** **5**a) generally uses a screen mask which includes a fabric mesh with the patterns of the image. The ink is pressed using a squeegee such that the ink penetrates the substrate through the portion of the mesh not covered with the fabric material, forming a printed pattern.^[^ ^158^, ^159^, ^160^ ^]^ Basically, this is a selective transfer process of ink through the open areas of the unmasked portions of a screen. Masking of the screen is accomplished by the transfer of a photographically produced image from its temporary film base support to the screen.^[^ ^161^ ^]^ Low cost and high processing speeds are some potential advantages of screen printing, however, changes in shear force can continually influence ink viscosity, leading to pattern distortion. Here, the printing resolution is limited by the printing speed. Thus, there is a need to optimize the formulation of conductive inks suitable for screen printing while maintaining the good conductivity and fidelity of printed patterns.^[^ ^160^ ^]^ One of the biggest advantages of screen printing is the versatility of the substrates including paper, paperboard, polymer materials, textiles, wood, metal, ceramics, glass, and leather. In addition, the screen printing process enables ink application not just to flat surfaces but to irregular ones too, as long as the thick ink adheres properly to the printed substrate and the screen can adapt to the substrate's shape consistently without distortion.^[^ ^162^ ^]^ The wide variety of polymer substrates requires different types of inks. Printing inks must be selected according to the type and surface characteristics of printing substrates. A sharp edge of printed image requires ink with higher viscosity in screen printing than in other printing techniques. Screen printing inks can be categorized by the drying process into the following groups: evaporative (water‐based and solvent‐based), oxidizing, catalytic, and UV inks. Solvent‐based inks are very common in polymer screen printing applications, but there are some other inks that are dried by a slower process of oxidation and polymerization, too. Few inks also use UV energy for curing by polymerization.^[^ ^162^ ^]^ Screen printing is by far the most widely used method for wearable e‐textile applications. Researchers have attempted screen printing for the fabrication of textile‐based strain,^[^ ^163^ ^]^ pressure,^[^ ^164^ ^]^ temperature,^[^ ^165^ ^]^ and humidity sensors.^[^ ^166^ ^]^ Several textile‐based biosensors such as an electrocardiogram (ECG),^[^ ^167^, ^168^ ^]^ electroencephalography (EEG),^[^ ^169^ ^]^ electromyography (EMG),^[^ ^170^ ^]^ and electro‐oculography (EOG)^[^ ^170^, ^171^ ^]^ were also reported via screen printing method. In addition to these, several screen‐printed textile‐based supercapacitors,^[^ ^172^ ^]^ heating elements^[^ ^144^ ^]^ were also demonstrated. We will discuss in detail about screen‐printed wearable e‐textiles in the subsequent sections.

### 3.2 Gravure Printing

Gravure is a widely used roll‐to‐roll printing method (Figure 5b), characterized by excellent print quality with high speed, often used for large volumes of magazine and catalog printing.^[^ ^174^, ^175^ ^]^ An engraved metal roll is used in gravure printing to transfer the ink directly to the substrate. The depression in the engraved surface forms the print pattern. The roll is immersed in the ink bath where the etched portions pick up and hold the ink followed by a blade to remove the excess ink. An impression cylinder pushes the substrate film with the depressed, ink‐filled regions of the gravure roll, thus transferring the ink to the film. A dryer after that drives off the solvent creating the final layer of coating.^[^ ^176^, ^177^ ^]^ The depressions in the gravure rolls, to hold the ink can be etched chemically, mechanically, or by laser. One of the advantages of gravure printing is that the rolls are hard, and durable, thus is cost‐effective for long runs though the initial costs are high. The printing quality depends on the ink viscosity, substrate speeds as well as the pressure of the impression roller. A relatively high print pressure (1–5 MPa) and quite low‐viscosity inks (50–200 mPa) perform a good ink transfer. However, comparatively lower viscosity inks (1–10 mPa) have also been applied successfully for the printing of some biomolecules.^[^ ^178^ ^]^ Another advantage is stretchy and unstable fabrics can also be printed by this technique efficiently.^[^ ^179^ ^]^ The printing rate in gravure printing may be achieved up to 25–1000 m min^−1^.^[^ ^175^ ^]^ Liquid gravure printing inks are dried by using physical method, i.e., by evaporation of the solvents, and inks of two components by chemical curing. The solvents speed up the drying process, but volatile organic chemicals (VOCs) are emitted, which need to be recovered (typically 87–98%). A small part of the remaining solvents (1‐3%) may still penetrate the pores of the paper, causing the characteristic smell of gravure printed products. Recent developments decrease the amount of residual solvents, substitute toluene, and minimize solvent emission. Water‐based inks are of increasing interest, due to no VOC emission, especially in the field of packaging materials. But more than threefold to fivefold energy is required in the drying unit than is required with solvent‐based inks, and this also involves print quality problems.^[^ ^174^ ^]^ Since gravure printing is widely used for large‐scale roll‐to‐roll textile batches, for lab‐scale e‐textiles it is not a very popular method. However, few researchers reported gravure printing for textile‐based humidity sensors.^[^ ^180^, ^181^ ^]^ We assume, upon commercialization, that gravure printing might be a high‐speed production choice for large‐scale e‐textile fabrication.

### 3.3 Flexographic Printing

Like gravure printing, flexographic (shortly named as flexo) printing is another high‐speed roll‐to‐roll printing technique, Figure 5c. The printing process involves transferring ink, from the surface of a flexible plate to the substrate. The pattern is made into plates that are attached to a roll. The printing ink is metered onto the surface of the plate by an “anilox”‐ an engraved/ etched roller of chrome or ceramic similar to a gravure cylinder but with a uniform distribution of cells (size, shape, and depth). The volume of ink to be transferred to the printing plate depends on this specification of the anilox. The ink is taken into these cells and a doctor blade assembly wipes the excess ink subsequently. The plate transfers the ink film from the anilox to the substrate by impression.^[^ ^182^ ^]^ Flexo plates use vulcanized rubber or photopolymer materials that are attached to rotating cylinders.^[^ ^183^ ^]^ The difference between flexographic printing the gravure printing is, that instead of relying on impressing the film into the ink containing cavities of the roll, the ink on the flexographic plates relies on the ridges of the pattern.^[^ ^176^ ^]^ The relatively inexpensive plates, advanced quality, and productivity of flexographic printing often make a printer difficult to choose between flexographic and gravure printing. Flexography is now dominant process for labeling, leaflets, and cartons markets in packaging.^[^ ^182^ ^]^ Flexographic printing allows to achieve increased printing speeds, of ≈10 m^2^ s^−1^. Resolutions of ≈0–100 µm can be achieved. This technique is also suitable for the creation of RF circuits, where very good conductivities are necessary since a thicker ink layer can also be deposited (5 µm).^[^ ^184^ ^]^ Flexographic inks maybe solvent‐based, water‐based, or UV‐curable inks. To ensure the regular ink flow in the printing unit, low‐viscosity printing inks are required in flexographic printing, generally lower than 0.05–0.5 Pa s with a flow time equal to 18–35 s. Concentrates of both solvent‐ and water‐based flexographic have relatively higher viscosity in the range of 0.1–0.25 Pa s.^[^ ^185^ ^]^ High‐solvent content and a low level of pigment concentration are required. An exception is for UV‐curable inks, which do not include any solvents. The basis for their production is binders, usually acrylates, which under the influence of UV radiation converted into a dried layer. In the case of solvent‐based and water‐based inks, drying of the ink on a printing base is the result of evaporation of the solvent. The more volatile solvents are in the ink, the quicker the drying occurs.^[^ ^186^ ^]^ Similar to gravure printing, flexography is suitable for large‐scale production. We assume for large‐scale production of commercial‐grade e‐textiles, flexography could be a suitable choice.

### 3.4 Inkjet Printing

Inkjet printing (IJP) also known as digital printing of functional materials with specific electrical, chemical, biological, optical, or structural functionalities has gained significant research interest due to its wide range of applications in different processes and purposes, from the batch coding of soft drink cans to smart electronic textiles.^[^ ^187^, ^188^ ^]^ IJP allows the deposition of tiny droplets onto the substrate without depending on the high‐speed operation of mechanical printing elements. Nozzle sizes for the printers are usually 20–30 µm with ink droplets as small as 1.5 pL with the achievement of high resolution (dots per inch).^[^ ^189^ ^]^ Besides 2D print results, in ink jet printing, “structural” fluids can also print layers that harden to form 3D structures. In spite of all these, several considerations such as the printing speed, cost‐benefit issues, printed film uniformity and material, and fluids' jet‐ability as ink‐ are still points of concern for printers.^[^ ^190^ ^]^ Two distinct modes of inkjet printing are there, 1) Continuous inkjet (CIJ), suitable for industrial scale and mass production, and 2) Drop‐on‐demand (DOD), used for small‐volume and prototype sample production.

### 3.5 Continuous Inkjet (CIJ) Printing

Continuous inkjet printing (CIJ) utilizes a pressurized fluid stream and a piezoelectric element at high frequencies (≈20 – 80 kHz), to form droplets, Figure 5d. A continuous and consistent stream of fluid droplets of uniform size and spacing can be generated by the careful adjustment of the voltage and frequency of the piezoelectric device. A conductive material placed in the fluid can impart an electric charge on selected drops breaking them off from the fluid stream which are further deflected by means of high voltage deflector plates to form various patterns onto the substrate. Uncharged droplets are captured by a gutter mechanism and re‐circulated through the system. A “catcher,” a plate situated below the nozzle to perform patterning, actuates into and out of the path of the droplets, only allowing the droplets to pass when pattern material is required.

For industrial environments, CIJ offers cheaper alternatives and a high print rate by minimizing transient and clogging issues, which is a limitation of DOD.^[^ ^191^ ^]^ CIJ printing process possesses high flexibility, precision, and speed allowing it to be used in applications in high‐speed graphical applications such as coding, marking, and labeling, as well as textiles and micro‐manufacturing industries. The benefits of CIJ over DOD are improved image quality, throw distance, and the ability to deflect droplets independent of gravity.^[^ ^192^ ^]^ Since CIJ printing produces large‐diameter droplets (≈40 mm), the print resolution is relatively low. They are able to use volatile solvent‐based inks, allowing rapid drying and proper adhesion on many substrates. This also makes the CIJ a messy and environmentally unfriendly technology.^[^ ^190^ ^]^ CIJ can be designed as a binary or multiple‐deflection system based on the drop‐deflection methodology. In a binary deflection system, the drops are either charged or uncharged. The charged drops can fly directly onto the media, while the uncharged drops are deflected into a gutter for recirculation. On the other hand, in a multiple‐deflection system, drops are charged and deflected to the media at different levels; the uncharged drops fly straight to a gutter to be recirculated. This approach allows a single nozzle to print a small image swath.^[^ ^193^ ^]^

### 3.6 Drop‐on‐Demand (DOD) Inkjet Printing

Drop‐on‐demand (DOD) printing, today, is the most commonly used printing technique in laboratory and small‐volume printers, Figure 5e. Due to the flexibility of the variety of inks used and the simplicity of application, it works very well with the prototype environments. The printer nozzle while passing over the substrate, an actuator ejects a droplet wherever patterned material is required,^[^ ^191^ ^]^ the production of each drop occurs rapidly in response to a trigger signal. Typically DOD print head contains multiple nozzles (100 to 1000, specialist heads may contain only a single), and instead of drop ejection resulting from external fluid pressure as in CIJ printing, the drop's kinetic energy derives from sources located within the print head, very close to each nozzle. In DOD printing, the liquid emerging from the print head in the form of a jet is then detached from the nozzle and collapses under surface tension forces to form one or more droplets.^[^ ^194^ ^]^ Typical drop volumes of individual ink drops in DOD are in the range of 1–70 pL and produce print spot sizes in the range of 10–50 µm in diameter. The slower speed (5000–20 000 Hz per second) of making drops is the limitation of DOD.^[^ ^195^ ^]^ The high accuracy and small droplet size of DOD inkjet printers are the key advantages for the direct patterning of functional materials. Depending on the way of generating the pressure pulse, the DOD inkjet printers can be further divided into four main types: thermal, piezoelectric, electrostatic, and acoustic. Most of the DOD ink‐jet printers, on the market, are using either the thermal or piezoelectric principle. Electrostatic and acoustic ink‐jet methods are yet to be developed for commercial wide‐scale application.

The thermal inkjet (often called TIJ) printer head is composed of a chamber that contains the print fluid. On one of the walls of the chamber, there is an electrical resistor designed to heat to high temperatures. Additionally, there is a nozzle to eject the print fluid. When current is applied to the resistor for a short span of time (a few microseconds), it heats the surrounding fluid in its immediate vicinity and causes local boiling (micro‐boiling, MB). The rapid pressure rise forces liquid at a distance from the resistor into the nozzle and out of the chamber. The rapid temperature rise and resulting temperature gradients on the resistor shorten its lifetime and therefore the lifetime of the entire head is shorter than other printer heads, such as the piezoelectric head.^[^ ^196^ ^]^ Though this is a simple and cheaper technology, there is a limitation of utilizing a range of print liquids (restricted to fluids that will satisfactorily vaporize).

Piezoelectric DOD works in a similar way to TIJ in that droplets are ejected by piezo‐ceramic distortion, which occurs when an electric field is applied to it. In a piezo DOD system, the actuator is based on a piezoelectric crystal, which changes shape as a consequence of a current being passed through it. In some print head designs, the piezo component pushes into the ink chamber to create pressure pulses, in others it surrounds the chamber or forms a wall of the chamber. The outer surface of the piezo‐ceramic has a conductive coating, that provides an electrical connection to it. The size of the ink droplets is determined by the voltage applied for piezo‐ceramic deflection, the pulse duration, and the diameter of the nozzle. Piezoelectric print heads can handle a wider range of liquids than thermal heads and the print heads have longer life. However, the print heads and supplementary hardware are costly.^[^ ^197^ ^]^

In the electrostatic inkjet printing method, the ejecting droplet through the nozzle is induced by electrostatic forces, applied between an electrode and the nozzle attracts the free charges within the ink toward its surface, the charged fluid is separated from the inkjet head as fine droplets. However, it is difficult to make the droplet smaller than the nozzle size in piezoelectric systems. To overcome this issue, the electrostatic inkjet method was introduced. Besides ejecting fine droplets, electrostatic inkjet printing is also able to print relatively viscous ink, offering higher resolution than that of piezoelectric inkjet printing.

The acoustic inkjet printing technology is a relatively recent development, where droplets are generated by acoustic energy. The ultrasound beam is focused with an acoustic lens onto the surface of fluid that produces an ink droplet. Print head nozzles are safe from clogging in this system with a versatility of ejected droplet sizes (ensured by changing the fluid‐to‐transducer distance in order to vary the focal spot diameter on the surface of the fluid).^[^ ^190^ ^]^

Inkjet printing delivers a small amount of materials as ink to a specific location of substrate, which opens the door to design versatility.^[^ ^190^ ^]^ It does not require stencils; achieving a cost‐effective printing choice for a much smaller print run than conventional screen printing.^[^ ^198^ ^]^ Being highly adaptive, it can be applied to a wide range of different processes and purposes, from batch to rapid prototyping in product design.^[^ ^187^ ^]^ It also provides possibilities for new workflows, short production runs, sustainable printing environments, quick response time, and customization.^[^ ^179^ ^]^ Considering all these benefits, inkjet printing is considered to be the future of e‐textiles. Therefore, after screen printing, inkjet printing is the most widely used technique to fabricate e‐textiles. Researchers have already reported inkjet printing methods to fabricate textile‐based strain,^[^ ^199^ ^]^ pressure,^[^ ^200^ ^]^ temperature,^[^ ^201^ ^]^ and humidity sensors.^[^ ^202^ ^]^ Several biosensors such as ECG,^[^ ^147^ ^]^ EEG,^[^ ^203^ ^]^ and EMG^[^ ^203^ ^]^ are also reported. Inkjet printing has also been used to fabricate textile supercapacitor electrodes.^[^ ^204^ ^]^

## 4 Functional Printing Inks

### 4.1 Formulation of Printable Inks

Conventional printable inks usually contain four components: Dye or pigment, binder, solvent, and additive. The main component colorant may either be a dye or a pigment, which needs to be dispersed properly in a vehicle, consisting of binders, solvents, and additives. All these combinedly provide viscosity and flow properties to the printing ink.^[^ ^205^ ^]^ Functional inks, on the other hand, have an electrically functional element instead of or along with the pigment. They usually possess electromagnetic, thermal, chemical, and/or optical properties, and are classified into conductive, semi‐conductive, dielectric, and resistive ink categories.^[^ ^206^ ^]^ Binder (also called thickener) holds the functional components and contributes to properties such as hardness, gloss, adhesion, and flexibility. They become an integral part of the pigment‐binder‐substrate system by crosslinking after evaporation (by drying or curing) is carried out of the printing ink. Alkyds, polyamides, polyimides, rubber, ketone, acrylic, epoxide, etc. resins are typical binders used in printing ink formulation. Solvents act as the diluent of the other ink components (i.e., pigments, binders, and additives). The main objective of using a solvent is to prevent the agglomeration of materials along with keeping the ink paste in fluid form, sufficient enough to apply on a substrate. For dissolving polar molecules, polar solvents such as water, alcohol, and esters are widely used. Liquid hydrocarbons are used for nonpolar molecules. Sometimes co‐solvents are combined with solvents to cut the cost. An ideal solvent and/or co‐solvent dissolves the resin/polymer without dissolving or degrading the pigment; evaporates at a compatible rate with the desired printing process, possesses required viscosity, and should be compatible with the image carrier. Depending on the printing processes, the substrates, the drying conditions, and the final purpose of the prints, water or any of a broad range of organic solvents can be used.^[^ ^173^ ^]^ However, the presence of residual toxic solvents in printed electronics still exists which limits the scalable adoption of wearable electronics.^[^ ^207^ ^]^ Therefore, for e‐textile application a solvent must make a stable solution with the functional ingredients,^[^ ^208^ ^]^ should not degrade the textiles themselves, and most importantly need to be non‐toxic and safe for physical health for everyday use.^[^ ^209^ ^]^ A water‐based ink system thus, could be considered to be beneficial to avoid such limitations for the e‐textile print ink formulation.^[^ ^210^ ^]^ Modifiers and/or additives are added to the print ink to either enhance the printability of the ink or of the cloth. Modifiers may tailor the viscosity or surface tension of the print ink to enhance the printing performance of the print ink. Several types of additives include preservatives (hygroscopic agents), anti‐oxidizing agents, fastness enhancers, or functional agents such as anti‐flammable agents, antiseptics, and disinfectants.^[^ ^211^ ^]^ Additives are incorporated into ink formulations in amounts not exceeding 5%,^[^ ^206^ ^]^ **Table** **2** presents a typical composition of various print inks.

**Table 2:** Typical composition of printable inks.^[^ ^173^ ^]^

Composition	Screen	Gravure	Flexographic	Inkjet
Pigment	12–20	12–17	12–17	5–10
Binder	45–65	20–35	40–45	5–20
Solvent	20–30	60–65	25–45	65–95
Modifier and/or additive	1–5	1–2	1–5	1–5

### 4.2 Requirements of Conductive Inks

As we discussed in Section 1, the development of electronic textiles hinges on the integration of electro‐active materials with textile substrates. The 1D conductive textiles could be typically formed by either spinning inherently conductive polymers or blending electroactive substances into the spinning solution. On the other hand, for 2D conductive fabrics, methods such as padding and coating are commonly employed, ensuring a thorough coverage of electroactive materials. Consequently, these processes involve substantial usage and disposal of conductive materials, often presenting issues with toxicity, environmental impact, and non‐biodegradability. However, an alternative approach to address these concerns is through printing, which involves the localized deposition of active materials in the form of printing ink or paste directly onto textiles. This method offers a sustainable solution, significantly reducing material wastage compared to traditional yarn and fabric coating processes. Moreover, printing techniques are known for their simplicity, cost‐effectiveness, time efficiency, eco‐friendliness, and versatility when applied to various textile substrates. A liquid to be considered as printing ink or paste must need to possess some attributes.

A liquid has an internal resistance to flow, “viscosity” is a measure of this resistance to flow or shear. It can also be termed as a drag force and is a measure of the frictional properties of the fluid.^[^ ^212^ ^]^ According to viscosity, printing inks may be classified as either low‐viscosity printing inks or high‐viscosity print pastes. Liquid printing inks like flexographic, gravure, and inkjet inks fall under the low‐viscosity category.^[^ ^185^, ^213^ ^]^ In the case of offset, screen printing, and pad printing inks, the viscosity remains >1 Pa‐s, which are categorized as high‐viscosity printing inks. The term “rheology”, a key feature to describe the print paste behavior, is “the study of the deformation and flow of matter”.^[^ ^214^ ^]^ For a print ink (for lower viscosity formulation, usually used for gravure, flexographic, or inkjet process), density, viscosity, surface tension, drying rate, flow behavior, flow time, etc. are important factors determining the efficient output of the print process. Though the viscosity of a print paste or ink can be modified; it is challenging to keep the same electrical properties with changing the viscosity. Increasing the temperature decreases the viscosity whereas the evaporation of solvent increases the viscosity. Solvent might be used to tune the viscosity of the ink. In addition to this, increasing the dispersant concentration decreases the viscosity of the ink.^[^ ^213^ ^]^

Surface tension of the ink is another important parameter of the print paste or ink, required for the formation of drops, which also affects the interaction between the ink and the substrate, i.e., wettability and printability. Surface tension depends on the composition; solvents such as polar liquids have higher and the nonpolar one has lower surface tension. Thus, water (surface tension 73 mN m^−1^) based inks have higher values, and ethyl alcohol (24 mN m^−1^) or other nonpolar solvents‐based inks have lower values of surface tension.^[^ ^215^ ^]^ A proper bonding exists between a liquid and a substrate surface when the surface tension of the liquid is 2–10 mN m^−1^ lower than the surface energy of the substrates.^[^ ^216^ ^]^ An increase in the temperature or an increase in solid content can decrease the surface tension of an ink.^[^ ^213^ ^]^

According to the viscous flow behavior, liquids may either be Newtonian fluids or non‐Newtonian fluids. The viscosity of a Newtonian fluid remains constant and they exhibit ideal viscous flow behavior. Any physical or chemical modification to the ideal Newtonian fluid may affect the flow behavior.^[^ ^217^ ^]^ Non‐Newtonian fluids are two types; Shear‐thinning or pseudo‐plastic fluids are characterized by an apparent viscosity, which decreases with increasing shear rate.^[^ ^218^ ^]^ The usual printing pastes exhibit shear‐thinning flow behavior.^[^ ^185^ ^]^ In the case of shear thickening (or dilatant) fluids, the apparent viscosity increases reversibly as the shear rate increases.^[^ ^219^ ^]^ Shear thickening fluids are not recommended in printing, since the fluid does not distribute properly, particularly with fast‐running presses related to the occurrence of the high shearing stresses.

Another characteristic is important for print ink named “thixotropy”. This is a time‐dependent phenomenon that means that the viscosity of the fluid depends on time as well as shear rate, i.e., the viscosity of thixotropic fluids decreases with time.^[^ ^220^ ^]^ When stirring at a constant shear rate, the gel structures in the ink break, and then a gradual recovery of the structure occurs, when the stress is removed, thus thickening when the ink is kept standing. The highly viscous printing inks, in most cases, are thixotropic; therefore, the viscosity of a printed ink is higher than when the ink is in the printing unit rollers.^[^ ^185^ ^]^

Particle size is also an important parameter for functional print ink. Decreasing the particle size increases the surface area and increases the amount of stabilizing agents required. It also results in a high surface‐to‐volume ratio, lowering the required sintering temperature. Small particle size and uniform size distribution in the ink produce higher viscosity inks and denser printed patterns; therefore, improving the functionality. Moreover, particle morphologies, e.g., nanospheres (NS) and nanowires (NW), also affect both the electrical conductivity and energy needed for sintering. Higher electrical conductivity can also be achieved by using inks with higher solid content. An increase in the solid content also leads to viscosity decrease under shear stress, allowing the ink to flow more smoothly from one surface to another while still preventing excessive spreading of the ink after printing. The rheological behavior of the ink can thus be tailored by changing the solid content of the ink.^[^ ^213^ ^]^ **Table** **3** provides a typical comparison of various print ink properties.

**Table 3:** Comparison among various printable ink parameters.^[^ ^185^, ^213^ ^]^

Ink viscosity	Print technology	Viscosity (Pa.s)	Surface tension (mN m^−1^)	Layer thickness (µm)	Feature size (µm)	Maximum particle size (nm)	Maximum preferred particle size (nm)	Maximum solid content (%)	Flow Properties
Low‐viscosity printing inks	Flexography	0.01–2	28‐38	0.04‐2.5	80	15 000	3000	40	Newtonian or non‐Newtonian shear thinning with a small deviation
	Gravure	0.01–1.1	41–44	0.1–8	70–80	15 000	3000	30	Non‐Newtonian
	Inkjet	0.001–0.05	25–50	0.05–20	20–50	1/10th of nozzle diameter	50	20	Non‐Newtonian
High‐viscosity printing ink (paste inks)	offset	20–100	30–37	0.5–2	10–50	10 000	1000	90	Non‐Newtonian, shear thinning, thixotropic
	Screen printing	0.1–1000	30–50	0.015–100	20–100	1/10th of mesh opening	100	90	Thixotropic

Printability (i.e., the ability to absorb inks or lacquers or other liquid and paste substances) of any substrate depends on its porosity, surface free energy (SFE), structure and dimension, durability, hydrophilic property, and optical properties. Besides these, the selection of ink with an appropriate level of surface tension is also necessary. It is generally believed that the surface tension of the ink should be lower than the surface free energy of the substrate. Printability of polymer substrates may be improved by increasing in surface free energy of the material (by any means of activation), by the reduction of surface tension of applied inks; and by the maximum reduction of the polar component of substrate SFE.^[^ ^215^ ^]^

Fluid properties such as viscosity and surface tension have an influence on the formation of droplets from an inkjet printer. The spreading behavior of the inks is determined by the hydrodynamic properties namely; the Reynolds number, (*Re* = *ναρ*/*η*, is the ratio of inertial to viscous forces), and the Weber number, (*We* = *ν2αρ*/*η*, is a balance between inertial and capillary forces).^[^ ^221^ ^]^ Both are combined to form the Ohnesorge number (*Oh*). The inverse of the Ohnesorge number is termed as printability. This is usually expressed by the *Z* number. The Ohnesorge number is given as √*We*/*Re* or,

(1) $$Oh=\frac{\eta }{\sqrt{\left(\gamma \rho a\right)}}$$

where *η* is dynamic viscosity, *γ* is surface tension, *ρ* is density and *a* is the characteristic length (usually the diameter of the print head's nozzle). The printability,

(2) $$Z=\frac{1}{Oh}=\frac{\sqrt{\left(\gamma \rho a\right)}}{\eta }$$

**Figure 6:**
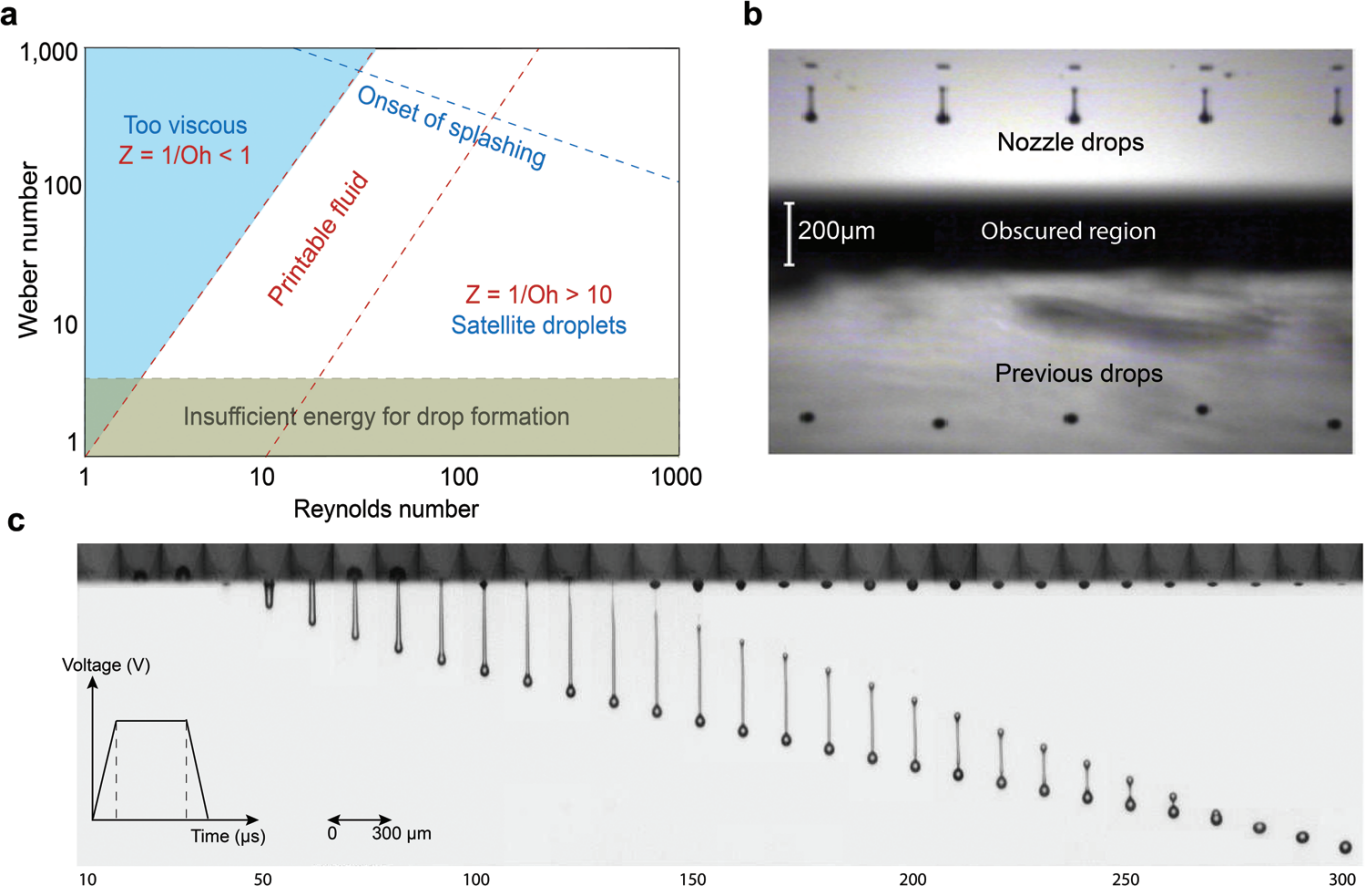
Inkjet Ink property requirement and drop formation. a) Graphical means of assessing ink suitability using nondimensional numbers. Reproduced with permission.^[^ ^223^ ^]^ Copyright 2012, Emerald Publishing Limited. b) Example of a drop watcher image on DMP‐2831, showing five nozzles jetting 3 wt./wt. % polyDADMAC solution with a 30 µs strobe delay. Reproduced with permission.^[^ ^224^ ^]^ Copyright 2020, The Author(s). c) Grayscale images of the ink jetting captured by the drop watcher system with the time sequence of the waveform. Reproduced with permission.^[^ ^225^ ^]^ Copyright 2022, The Authors.

Such a fluid velocity‐independent, dimensionless number has advantages as a suitable metric for fluid selection. Fromm suggested that *Z* > 2 for stable drop formation because viscous dissipation prevents drop ejection at lower values. Reis and Derby proposed, on the basis of computational fluid dynamics (CFD) modeling, that *Z* should be in the range 1 < *Z* < 10, with viscous dissipation preventing drop ejection when *Z* < 1, while satellite drops were predicted to form together with the primary drop when *Z* > 10.^[^ ^222^ ^]^ It was also found that *Z* has an influence on droplet volume too. As *Z* increases there is an increase in volume. Moon and co‐workers have said that *Z* should be between 4 and 14 for an ink to be printable.^[^ ^197^ ^]^ Jang et al. observed inks were easily ejected by the applied pressure without significant viscous dissipation when *Z*>14. The primary droplet fell with a high relative travel velocity so that the separated tail which formed transient satellites could not catch up with the droplet head. The large oscillatory kinetic energy and the high surface tension tend to induce a secondary rupture, which generates a primary droplet and permanent satellites. So fluids with *Z*>14 are not printable fluids.^[^ ^221^ ^]^ Considering the drop generation, drop flight, and drop impact, the optimal value of the physical condition for a robust DOD inkjet printing is typically with surface tension lying in the range of 20–50 mN m^−1^ and viscosity within the range of 2–20 mPa.s. This range broadly meets the criteria of a suitable ink for DOD inkjet printing to achieve high‐resolution print on the desired trajectory, whereas a narrower and specified range would be more applicable for specific print heads.^[^ ^49^ ^]^ **Figure** **6**a shows the ink properties suitable for inkjet printing. Figure 6b displays a drop watcher image showing five nozzles jetting 3 wt./wt. % polyDADMAC solution with a 30 µs delay. Figure 6c shows a grayscale image of ink jetting captured by the drop watcher system with the time sequence of the waveform.

## 5 Applications of Printed E‐Textile

### 5.1 Printed Textile Sensors

Wearable monitoring systems can be used for continuous physiological data monitoring which makes it a promising and effective technology for treatment/care plans for patients or elderly people, asses injury recovery or performance of sports players, etc.^[^ ^226^ ^]^ Sensors, with their ability to observe changes in their environment at any event, can provide their corresponding output, based on optical or electrical signals.^[^ ^227^ ^]^ Various types of sensors are available for wearable applications, namely motion sensors, biological sensors, and environmental sensors. Wearable motion monitoring systems can be used for navigation, and man–machine interaction, i.e., to detect motion‐relevant sensor information, such as posture and position. Gyroscopes, accelerometers, and geomagnetic sensors are a few common motion sensors. Biosensors, a rapidly evolving technology, are composed of biological materials and receptors and act as advanced detectors in biotechnology. They can detect and monitor vital signs and predict early signs of diseases, such as blood pressure sensors, blood glucose sensors, ECG sensors, temperature sensors, etc. Environmental sensors measure relevant indicators in the environment and carry out weather forecasts and health warnings. They also explore the influence of environmental factors on experimental samples in scientific research. This includes temperature and humidity sensors, UV sensors, soil acidity or alkalinity sensors, light sensors, etc.^[^ ^228^ ^]^ Sensors, the core of such wearable monitoring equipment, inevitably contact with the skin but can cause several problems, such as discomfort due to the presence of sensory nerves and sweaty skin. Besides affecting wearer comfort, a conventional rigid sensor may suffer from the signal error of some motion artifacts due to its insufficient flexibility and adaptability. Existing wearable devices are stiff, low precision, and consume high power due to the characteristics of sensing elements and therefore, the development of new flexible sensors is of great significance to meet those challenges.^[^ ^229^ ^]^

Sensors are devices able to detect external stimuli and convert them into standardized signals. Conventional sensors are usually rigid and cannot be deformed readily. Flexible sensors, in contrast, can easily be attached to various surfaces and can be used in wearable and portable electronics. As a result, they enable applications in electronic skin, robot sensing, wearable health monitoring, and so forth. Printed electronics (PE) are a simple but promising aspect in this regard to produce a wide range of electronic circuits and sensor devices on various flexible substrates^[^ ^157^, ^230^ ^]^ Available printing techniques include screen, gravure, inkjet, and even 3D printing technologies have been utilized to produce low cost, light weight, large‐area, biocompatible and flexible electronic sensor devices such as electrochemical sensors,^[^ ^231^, ^232^, ^233^ ^]^ enzymatic sensors,^[^ ^234^, ^235^ ^]^ pressure sensors,^[^ ^236^, ^237^ ^]^ and strain sensors,^[^ ^238^, ^239^ ^]^ etc. Among the several printing technologies, screen‐printing is a well‐established but cheap technique that has been exploited commercially for the fabrication of bio‐ and chemical sensors.^[^ ^240^ ^]^ However, most of the existing flexible and stretchable sensors can detect or monitor only one single stimulus at a time.^[^ ^241^ ^]^ To make up for this drawback and to expand the capacity of sensing applications, recent research has been targeting the development of various multifunctional sensors that can detect multiple stimuli simultaneously or separately.

### 5.2 Sensor Working Principle

**Figure 7:**
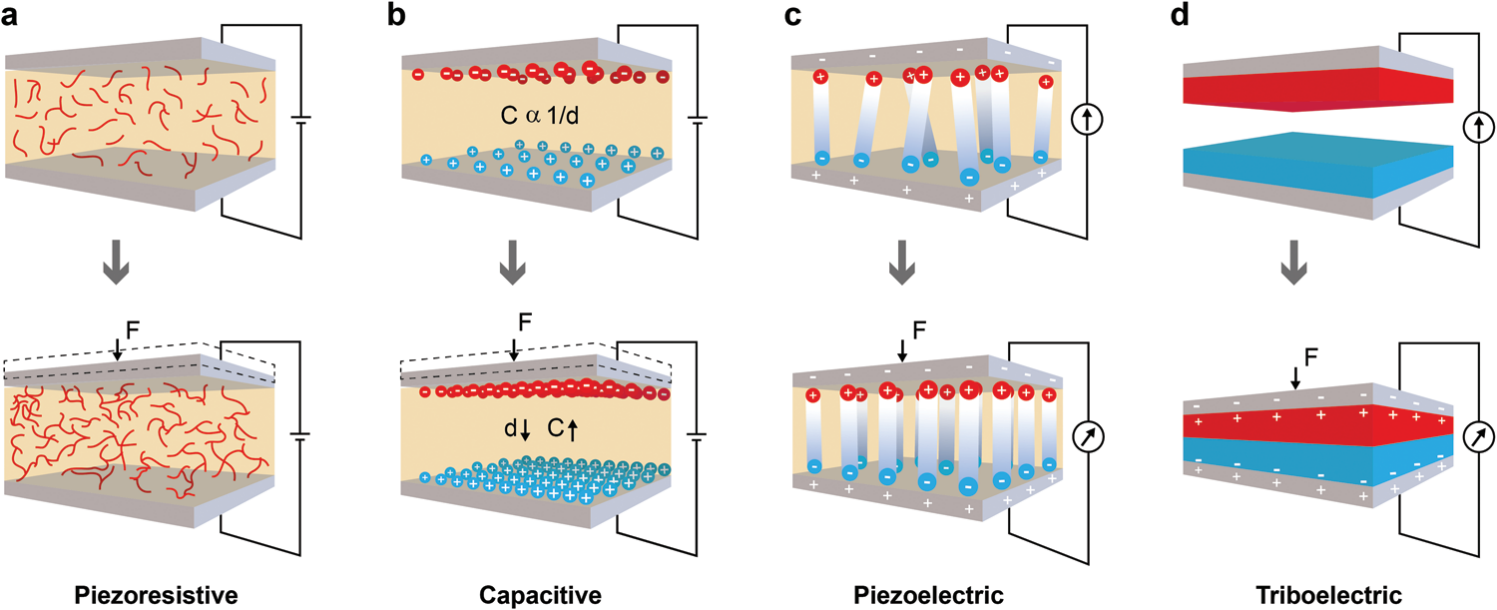
Working principle of printable textile sensors, a) Piezoresistive, b) Capacitive, c) Piezoelectric, and d) Triboelectric sensor.

Sensors could be classified as either active or passive sensors. Active sensors can convert the input energy into a measurable difference in potential (V or sometimes I) without any external power supply, however, passive sensors require an external power supply to convert physical stimuli into electrical forms (R, C, L, I). The majority of the textile sensors so far have relied on passive sensing (especially the resistance changes).^[^ ^242^, ^243^ ^]^ As per the transduction mechanism, the sensors could again be classified as piezoresistive, capacitive, piezoelectric, triboelectric, inductive, electromagnetic, and photoelectric etc., however, the first four are the most used sensors.^[^ ^244^, ^245^ ^]^ **Figure** **7**.

The working principle of a piezoresistive sensor is the change of corresponding resistance of the sensor due to the change in the applied pressure from an external source.^[^ ^246^ ^]^ The simple structure, working mechanism, ease of fabrication, and excellent performance of such sensor make it popular and thus being studied widely. A resistive sensor is, a conductive network of active materials serving as a resistor under an applied voltage, i.e., the electrical resistance of the conductive network changes as a function of the applied mechanical strain. The variation in the resistance originates from the geometrical changes (i.e., length and cross‐sectional area), the intrinsic resistive response of the active materials, the tunneling effect, and/or disconnection mechanism during stretching. While releasing the tensile/compressive strains, the resistance recovers to its initial values in a reversible manner, the deformation state can, therefore, be readily measured by recording the electrical resistance changes.^[^ ^247^ ^]^ Conducting fibers, yarns, and/or textiles can be utilized as a resistor when placed in an electronic circuit as well as a sensor device. Sensing functionality can then be achieved in the dynamic mode by measuring changes in the resistance *R*:

(3) $$R=\rho \frac{L}{A}$$

where *ρ*, *L*, and *A* are the resistivity, length, and average cross‐sectional area, respectively. The resistance variation that occurred due to the dimensional change is termed piezo resistivity and is widely studied for its potential application as strain or pressure sensors.

For evaluating the sensing performance and selecting the appropriate piezoresistive physical sensors suitable for specific applications, a few parameters are considered such as sensitivity, stretchability, durability, linearity, selectivity, detection limit, response time, transparency, etc.^[^ ^248^ ^]^ Sensitivity (*S*) is a value to measure the ability of sensors to convert external stimuli into electrical signals.^[^ ^249^ ^]^ For strain sensors, gauge factor (*GF*) is popularly used to evaluate the sensitivity determined by the ratio of normalized resistance change, ∆*R*/*R*_0_, to the applied strain, ɛ.^[^ ^248^ ^]^

(4) $$GF=\frac{\Delta R/R_{0}}{\varepsilon }$$

For a pressure sensor, if ∆*P* is the pressure variation, the sensitivity is then defined as:

(5) $$S=\frac{\Delta R/R_{0}}{\Delta P}$$

Again, for a thermistor, the sensitivity is usually given by the temperature coefficient of resistance, α, which is determined by the ratio of relative resistance change, *dR*_T_/*R*_T_, to the temperature variation, *dT*.^[^ ^248^ ^]^

(6) $$\alpha =\frac{1}{RT}\frac{dRT/RT}{dT}$$

where *R*_T_ is the resistance of the thermistor at a given temperature *T*.

The stretchability of a sensor is regarded as a measure of the maximum tensile strain that the sensor can sustain with stable sensing performance under repeated loading and unloading. The durability is the capability of the sensor to maintain stable and reliable electrical functionality and mechanical integrity under long‐term continuous loading–unloading cycles. Many factors, including microstructural changes, oxidation or corrosion of sensing materials, and environmental influences, can affect the durability of a sensor. The linearity of resistance response is another important parameter of sensors because nonlinearity makes their calibration complex and difficult. Easy calibration is essential to provide meaningful numerical readings with good repeatability and resolution. The detection limit is a measure of the smallest quantity with a specified precision or reproducibility of a sensor. For example, the detection limit for carbonized silk‐based strain sensors and pressure sensors is ≈ 0.01% strain and 0.8 Pa, respectively.^[^ ^248^ ^]^ The hysteresis of conventional metal‐based sensors is often caused by a combination of mechanical and temperature hysteresis, whereas the viscoelastic nature of polymers and the interactions between the nanomaterials and polymer substrates are the main sources of the hysteresis of stretchable sensors. The response time is defined as the time needed for a measurable response in the steady state.^[^ ^248^ ^]^ In addition, optically transparent sensors that are “invisible” and “unfelt” are urgently needed for next‐generation wearable sensors because such sensors can be worn on the user's skin without affecting daily activities. These transparent and elastic sensors can be integrated or combined with other components to design skin‐like multifunctional electronic devices.^[^ ^248^ ^]^ Piezoresistive pressure sensors can measure statically as well as dynamically with less susceptibility to noise. Their electronics are less complex than those of the sensors. Furthermore, piezoresistive sensors can be produced within a printing process, making them mass‐producible, low‐cost, and adjustable with regard to spatial resolution.

A capacitive sensor, on the other hand, shows a change in its capacitance when pressure is applied from an external source. Bearing the principle of a parallel‐plate capacitor, a dielectric material is sandwiched between plates. With the application of an external force, the distance between plates is altered and a change in capacitance is observed. Capacitive sensors are highly sensitive and very responsive with a wide dynamic detection range.^[^ ^250^ ^]^ Capacitive pressure sensors can operate statically as well as dynamically, and their measurements are highly reproducible. They are sensitive to moisture and comparatively cost‐intensive to manufacture due to a necessary complex filter system in the electronics to reduce noise. They introduce time‐dependence and oscillatory electrical behavior.^[^ ^136^ ^]^ If d is the distance between electrodes of area *A*, *ε*_r_ is the relative permittivity of the dielectric, and *ε*_0_ is the permittivity of vacuum, the capacitance, *C* for a parallel plate capacitor is determined by):

(7) $$C=\varepsilon_{r}\times \varepsilon_{0}\frac{A}{d}$$

Piezoelectric sensors are mainly composed of piezoelectric‐sensitive materials, able to convert mechanical energy to electric energy and vice versa. When external pressure is applied to deform the material, positive and negative charge separation occurs within the functional material on the two opposite surfaces of the material, forming a potential difference inside. The potential difference is examined to determine the effect of external forces.^[^ ^228^ ^]^ Piezoelectric pressure sensors do not need any external power supply and can be measured with high sensitivity. They are less sensitive to temperature influences, but they require complex metrological analysis and are incapable of measuring statically.

The triboelectric sensors convert random mechanical energy into electrical signals without consumption of power while still exhibiting outstanding sensitivity,^[^ ^251^ ^]^ by utilizing the coupling effect of triboelectrification and electrostatic induction.^[^ ^252^ ^]^ Triboelectricity is a kind of contact electrification, when the surfaces of two materials with different electron affinities are in contact, they get surface charges of different polarities, electrons transit from a high energy level to a low energy level, converting kinetic energy into electrical energy. As the external kinetic energy causes the two triboelectric materials to produce periodic motion, the induced potential difference between the electrodes also changes periodically. When the load is connected, periodic alternating current is generated, which can then be used as an energy harvester to convert kinetic energy into electrical energy.^[^ ^253^ ^]^

#### 5.2.1 Printed Textile Strain Sensors

Strain sensors can detect deformations or structural changes occurring in infrastructures and are thus used potentially in various applications such as human motion detection, damage detection, characterization of structures, and exhaustion studies of materials in robotic systems, prosthetics, healthcare, and flexible touch panels.^[^ ^254^ ^]^ A strain sensor typically consists of a conductive pattern able to reflect a change in the electrical read‐out upon geometric deformation.^[^ ^173^ ^]^ Several painted(PET),^[^ ^255^ ^]^ coated^[^ ^256^, ^257^ ^]^ or printed sensors were reported on KAPTON (Inkjet),^[^ ^258^ ^]^ plastic (PEN) (screen print),^[^ ^259^ ^]^ PI (screen print),^[^ ^260^ ^]^ paper (screen print)^[^ ^261^ ^]^ or silicon^[^ ^254^, ^262^ ^]^ or silicon elastomer (screen print),^[^ ^238^ ^]^ (inkjet print).^[^ ^263^ ^]^ Textile has also been investigated as a promising substrate for printed sensor fabrication. **Table** **4** summarizes the printed textile‐based strain sensors.

**Table 4:** Summary of printed textile strain sensors.

Method	Sensing mechanism	Substrate	Composed of	Sensitivity	Stability	Reference
Screen printing	Piezoelectric	Plain warp‐knitted, 78% PET, 22% elastane fibers (2015)	PEDOT:PSS printed conductive patch with melt‐spun piezoelectric poly(vinylidene fluoride) (PVDF) fibers yarn			[264]
	Piezoresistive	Cotton, Polyester, Cotton/polyester (50/05)‐woven, Cotton Knit, Polyester Nonwoven (2018)	Iron oxide, Cobalt, Silver, Single‐walled carbon nanotubes, Multi‐walled carbon nanotubes			[163]
	Piezoresistive	PU‐based stretchable fabric (2021)	Polyurethane (PU) elastomer, CB nanoparticles, poly(3‐hexylthiophene‐2,5‐diyl) (P3HTs), and PDMS microbeads	GF of 57	Wide sensing range (≈130%), excellent repeatability (>10 000 cycles), waterproof capability (contact angle ≈112)	[265]
	Piezoresistive	Cotton (2022)	Graphene			[151]
Inkjet printing	Capacitive	PET cylindrical fabric (2015)	Silver and Parylene (dielectric layer)	Capacitance change of 0.7% at 1% of strain		[199]
	Piezoresistive	PET (2021)	Graphene‐PEDOT: PSS conductive ink	Gauge factor (GF) 165, with linear output signal at strain range 0–0.33%		[266]

Rajala et al.^[^ ^255^ ^]^ fabricated printed piezoelectric sensors on a flexible PET substrate with solution‐processed piezoelectric poly(vinylidenefluoride‐co‐trifluoroethylene) P(VDF‐TrFE) ink as an active layer. Evaporated silver on PET was used as the bottom electrode and the painted Ag glue as the top electrode. The fabricated sensors were characterized in normal and bending mode setups. Normal‐mode sensitivities showed values up to 25 pC/N, whereas bending‐mode sensitivities showed remarkably high values up to 200 nC/N. Åkerfeldt et al.^[^ ^264^ ^]^ also reported a piezoelectric sensor fabricated by using screen printing technology. Melt‐spun piezoelectric PVDF fibers with conductive cores were machine embroidered onto a textile glove to function as a sensor element. Electrodes and electrical interconnections were constituted by a screen‐printed conductive PEDOT:PSS formulation. A repeated strain of 10% only influenced the resistance of the interconnections initially and to a very limited extent. They also used the sensor data from the glove successfully as input to a microcontroller running a robot gripper, demonstrating its potential applications. In our previous study,^[^ ^151^ ^]^ we printed graphene on cotton textiles to form a piezoresistive sensor for monitoring human motion activity at different body parts.

**Figure 8:**
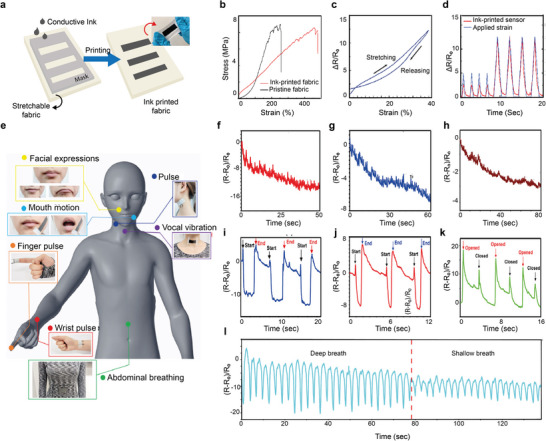
Printed textile strain sensors a) A schematic illustration for the process of inkjet printing bead‐blended composite ink onto a stretchable fabric. The inset shows a photograph picture of the ink‐printed composite fabric, and b) Stress versus strain curves for tensile deformation on pristine and ink‐printed composite fabrics. c) A hysteresis test of the ink‐printed textile sensor, d) Time‐dependent electrical responses of the ink‐printed textile sensor with repeated strains of 20% and 50%. Reproduced with permission.^[^ ^265^ ^]^ Copyright 2021, Elsevier Ltd. Applications of the wearable graphene textile strain sensors for detecting various subtle human motions: e) A schematic illustration of the sensor attached to different parts for detecting subtle human motions, f) The signal of finger pulse g) The signal of pulse h) Wrist pulse signal i) The signal of laughing, j) The signal of crying, k) The relative resistance changes during open and close mouth, and l) The detection of respiration rate with different breath modes. Reproduced with permission.^[^ ^257^ ^]^ Copyright 2018, American Chemical Society.

Jang et al.^[^ ^265^ ^]^ proposed a printed micro‐structured textile strain sensor with high sensitivity and durability. A composite ink, composed of a conductive mixture of elastic microbeads and conducting polymer, was incorporated into the sensor via the inkjet printing process. A microcrack structure on the textile strain sensor, yielding sensitivity with a maximum GF of 57 was developed, **Figure** **8**a–d. The printed strain sensor retained high sensing performance with a wide sensing range (≈130%), excellent repeatability (>10 000 cycles), and waterproof capability (contact angle ≈112). The sensor was further applied to an integrative user‐interface device, which monitored the respiration and arm motion signals in real‐time under both dry and wet environments. Quintero et al.,^[^ ^199^ ^]^ reported inkjet‐printed capacitive strain sensors on PET fibers for integration into textiles at a large scale. 10‐meter‐long functionalized PET fibers were woven with metallic interconnect fibers using a large‐scale industrial weaving machine which resulted in a 1 m^2^ smart textile demonstrator. Sensor measurements were performed for strains up to 1% of applications foreseen in predictive maintenance of industrial textiles and in the automotive industry. Kang et al.^[^ ^266^ ^]^ reported a flexible strain sensor fabricated by inkjet printing technology of graphene‐PEDOT:PSS conductive ink on a PET substrate. A high GF value of ≈165 of three high resistive (HR) paths was obtained with a linear output signal at the strain range from 0 to 0.33%.

In addition to conventional screen printing, the latest digital inkjet printing and 3D printing was also employed to fabricate piezoresistive sensor. Alaferdov et. al.^[^ ^267^ ^]^ proposed a new but simple and scalable method for the fabrication of a piezoresistive wearable strain and bending sensor. The sensor was based on a high aspect ratio (length/thickness ≈10^3^) of graphite nanobelt thin films deposited by a modified Langmuir‐Blodgett technique onto flexible polymer substrates. High response stability for >5000 strain‐release cycles and a device power consumption as low as 1 nW were achieved. Though not printed, graphene‐textile strain sensor was also reported to show tremendous potential for wearable electronic applications. The strain sensor exhibited a distinctive negative resistance variation with increasing strain with high sensitivity, long‐term stability, and great comfort. The sensor was demonstrated to detect various human motions such as bending the wrist, while writing English letters, bending a finger at a certain angle, bending the elbow and knee joint at various angles, during walking and running at different frequencies. Interestingly, the sensor was also able to detect various subtle human motions including signal of finger pulse, signal of a pulse, wrist pulse signal, signals of laughing, crying, during opening and closing of the mouth, and even at different breathing modes, Figure 8e–l. Though not on textiles, 3D printing of PDMS/MWCNT nanocomposite was also reported for realizing strain sensors.^[^ ^268^ ^]^

#### 5.2.2 Printed Textile Pressure Sensors

Flexible electronics have been playing an important role in improving the well‐being and quality of life in the form of electronic skin, human–machine interaction interface, physiological signal monitoring, etc.^[^ ^249^, ^269^ ^]^ Pressure sensors that can sense and convert pressure input into electrical signals,^[^ ^245^ ^]^ are widely applied in various new electronic equipment due to their distinct characteristics with high flexibility, high sensitivity, and light weight.^[^ ^228^ ^]^ Intraocular, intracranial, and cardiovascular pressure; cumulatively called physiological pressure, is a key parameter for the assessment of human health providing opportunities for early diagnosis of disease, such as lower limb problems, muscle rehabilitation, and wound monitoring, personalized therapy, and preventive healthcare etc.^[^ ^270^ ^]^ Pressure and touch sensors are able to detect physical pressure, physical contact, and even proximity. They represent an interactive technology widely applied in consumer portable devices such as smart phones or touch pads. Pressure sensors can be created via structures where an active sensing layer is “sandwiched” between contacts, or where the active sensing spots are connected to contacts or a combination of both.^[^ ^173^ ^]^ Researchers have reported various fabrication technologies for realizing textile‐based pressure sensors such as conductive Shieldex Nora‐Dell woven fabric,^[^ ^271^ ^]^ MXene coating of cotton fabric,^[^ ^272^ ^]^ dip coating of conductive woven fabric in ZnO solution,^[^ ^273^ ^]^ immersion of textiles in conductive GO solution^[^ ^257^ ^]^ or painting^[^ ^274^ ^]^ or printing on several flexible substrates, i.e., PET^[^ ^164^ ^]^ etc. As our review scopes only the printing techniques employed, **Table** **5** presents a summary of the textile‐based printed pressure sensors.

**Table 5:** Summary of printed textile pressure sensors.

Method	Sensing mechanism	Substrate (Reporting year)	Composed of	Sensitivity	Response time	Detection limit	Stability	Reference
Screen printing	Piezoresistive	Cotton (2018)	Ag NWs and Ag paste ink	2.46 × 10^4^ – 5.65 × 10^5^ / kPa in a wide pressure range (<30 kPa),	6/16 ms	0.76 Pa	> 41 000 loading– unloading cycles	[275]
	piezoresistive	Cotton (2019)	Carbon black (CB) from diesel soot, Ag paste, PEDOT:PSS	High sensitivity (81.61 kPa^−1^ in the range of 0–10 kPa), ultrawide workable pressure regime (0–100 kPa)	Rapid response and relaxation time (6 and 30 ms, respectively)			[276]
Screen and inkjet printing combined	Piezoresistive	Cotton & polyester (2021)	Carbon‐PEDOT: PSS nanocomposite solution, Ag nanoparticle ink	1.45/kPa	≈30 ms	150 gm		[200]

**Figure 9:**
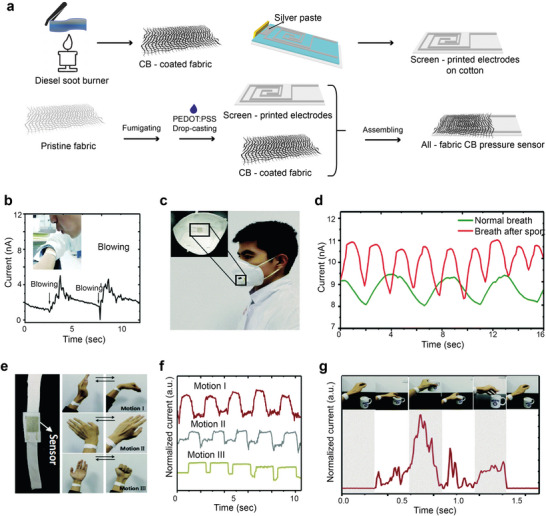
Printed textile pressure sensors a) Schematic illustration of the fabrication of the CB pressure sensor. Reproduced with permission.^[^ ^276^ ^]^ Copyright 2019, WILEY‐VCH. The real‐time current response to different mechanical forces using our textile‐based sensor: b) Digital photographs and the current curve for detecting the wind blowing from the mouth, c) The device was directly attached to the air outlet of the mask, d) The current signal responds to respiration under before and after exercise, e) Photographs showing that the flexible detector is fixed on the wrist by an elastic spandex band, and the human motion of bending the wrist (Motion I), waving (Motion II) and holding fist (Motion III), f) Real‐time current response corresponding to different human motions, g) The current signal in response to the whole process of lifting and lowering the cup. Reproduced with permission.^[^ ^275^ ^]^ Copyright 2018, The Royal Society of Chemistry.

Liu et al.^[^ ^276^ ^]^ used CB from diesel soot, an air pollutant produced during incomplete combustion of hydrocarbon fuels, as the active material to fabricate a high‐performance flexible all‐textile pressure sensor. A dry pristine nonwoven fabric was fumigated at different times over the diesel lamp. PEDOT:PSS and ethyl alcohol were well‐mixed and drop‐cast to prepare the conductive fabric. Screen‐printing method was adopted to print the commercially conductive silver paste on the precleaned cotton to cover the top CB‐coated fabrics to form the sensor, **Figure** **9**a. The pressure sensor exhibited outstanding performances, including high sensitivity (81.61 kPa^−1^ within the range of 0–10 kPa), extra wide workable pressure regime (0–100 kPa), rapid response, and relaxation time (6 and 30 ms, respectively). Zhou et al.^[^ ^275^ ^]^ designed a novel all‐fabric piezoresistive pressure sensor with a bottom interdigitated textile electrode screen‐printed with silver paste and a top bridge of AgNW‐coated cotton fabric. Benefiting from the highly porous microstructure, large surface roughness and ultra‐low resistance of the conductive fabric, the piezoresistive pressure sensors showed excellent detection performance, including an extra‐high sensitivity of 2.46 × 10^4^ kPa^−1^ to 5.65 × 10^5^ kPa^−1^ over a wide pressure regime (0–30 kPa), a giant on/off ratio of ≈10^6^, a fast response time (6 ms), and a low detection limit (0.76 Pa), Figure 9b–g. In addition, with the ability to detect various tiny signals of the human body, they demonstrated the devices to play the piano and computer games.

Khan et al.^[^ ^200^ ^]^ presented a combined screen‐inkjet printing fabrication route to develop a pressure‐sensing patch by utilizing conductive cotton fabric sandwiched between two parallel electrodes. A conductive nanocomposite solution by mixing carbon‐based paste and PEDOT:PSS was used. A nanofibrous stretchable cotton fabric was impregnated in the nanocomposite solution, which was used as the pressure‐sensing layer. The metallic plates and interconnect lines were developed by inkjet printing of Ag nanoparticles‐based solution on a PET substrate. The sensing blocks were patterned using screen‐printing. The thermally sensitive resin on one side of PET substrate enabled the sandwiching of the impregnated fabric through lamination. The produced piezoresistive sensors recorded resistance variation for as small as 1 gm weight. In addition to screen printing and inkjet printing, Li et al.^[^ ^277^ ^]^ reported the structural design of 3D flexible wearable pressure sensors using conductive polymer composites, exhibiting excellent piezoresistive performance, such as adjustable GF of 13.70–54.58, exceptional durability, and stability.

#### 5.2.3 Printed Textile Temperature Sensors

Measurement of temperature is a physiological indicator of health pathology.^[^ ^278^ ^]^ Continuous body temperature monitoring for patients facilitates healthcare providers to remotely track patients’ temperatures, promptly detect fevers, and thus allow timely intervention to prevent critical situations.^[^ ^279^, ^280^ ^]^ Flexible temperature sensors are therefore beneficial for real‐time temperature monitoring in healthcare, disease diagnosis, and ambient environment detection.^[^ ^281^ ^]^ Numerous flexible temperature sensors are already available in the market, however, most of them have not been integrated in a concealed manner and are not feasible for wearable applications due to the inability to bend, drape, or shear.^[^ ^282^ ^]^ The utilization of sensors, able to monitor skin temperature, concealed within everyday textile garments, could therefore greatly benefit patients and healthcare personnel.^[^ ^282^ ^]^ Surfaces able to distinguish spatial and temporal changes in temperature are critical for not only flow sensors, microbolometers, or process control but also future applications like electronic skins and soft robotics.^[^ ^283^ ^]^ Epidermal temperature sensors, already presented in studies, are mainly fabricated using a traditional photolithography process with several intermediate stages creating a substantial amount of material waste. Printed sensors thus offer great potential due to their flexibility, waste reduction, and low‐cost fabrication.^[^ ^284^ ^]^

The microstructure of any conductive network dominates the electrical behavior of the conductive material during the temperature change process. Several conductive materials including carbon nanotubes, graphene, conductive polymers, or metal nanostructures have been utilized to prepare temperature sensors.^[^ ^285^ ^]^ Temperature sensors could be resistance temperature detectors (RTDs), thermocouples, or thermistors.^[^ ^286^ ^]^ The RTDs use the temperature dependence of the material on electrical resistance to determine the temperature. The increase in temperature causes an increase in resistance due to the electron vibration at a higher temperature preventing the free flow of electrons in conductive materials.^[^ ^286^ ^]^ A high degree of accuracy, linearity, and quick response properties of RTDs, make them more preferable than thermocouples.^[^ ^286^ ^]^ RTDs are usually constructed with metallic material (such as platinum, nickel, copper, etc.) and thermistors are generally constructed with semiconductor materials. RTD‐based temperature sensors can measure big range of temperature changes (−230 °C to 660 °C) whereas thermistor‐based temperature sensors have limitations in the temperature range (−100 °C to 300 °C). Thermistors are generally less resistive than RTD‐based temperature sensors and can provide fast response, i.e., a small change in temperature can be measured with high accuracy. Due to construction with the semiconductor materials, there are 2 temperature coefficients of thermistor; positive and negative (PTC and NTC respectively). NTC thermistors are widely used as inrush current limiters, and temperature sensors, while PTC thermistors are used as self‐resetting overcurrent protectors, and self‐regulating heating elements. Most thermistors have NTC, i.e., their resistance value reduces with the increment in temperature. The sensitivity (S) and temperature coefficient of resistance (TCR) of the temperature sensors can be calculated by:

(8) $$S=\frac{R_{f}-R_{i}}{\Delta T}$$

(9) $$\mathrm{TCR}=\frac{R_{f}-R_{i}}{R_{f}\Delta T}$$

where *R*_f_ and *R*_i_ denote the resistance value obtained at temperature f °C and i °C respectively, Δ*T* is the change in temperature. The definition of TCR is the resistance change factor per degree celsius of temperature change. Though other printing techniques such as stencil^[^ ^287^ ^]^ and transfer^[^ ^288^ ^]^ have been reported, screen printing is most widely used to fabricate temperature sensors on various substrates such as PEN,^[^ ^289^ ^]^ PET,^[^ ^165^, ^281^, ^289^, ^290^, ^291^, ^292^, ^293^ ^]^ PI,^[^ ^283^ ^]^ PI‐PET^[^ ^294^ ^]^ and PDMS. **Table** **6**. summarizes the reported temperature sensors on various textile substrates.

**Table 6:** Summary of the printed textile temperature sensors.

Method	Substrate (Report year)	Composed of	Temperature Range	Sensitivity	Performance	Reference
Screen printing	PET, paper, textiles (2019)	Flake graphite (FG)/carbon nanotube (CNT)/ polydimethylsiloxane (PDMS) composite films (FG/CNT = 4:1)	40–80 °C	TCR 0.028/K		[165]
Inkjet printing	Taffeta fabric (2021)	Carbon nanotube (CNT) and PEDOT: PSS‐based ink	Room temperature to 50 °C	0.15%/°C for CNT, 0.41%/°C for PEDOT:PSS, and 0.31%/°C for CNT/PEDOT:PSS	Bending stability with a resistance change of 0.3% up to 1000 cycles	[295]

**Figure 10:**
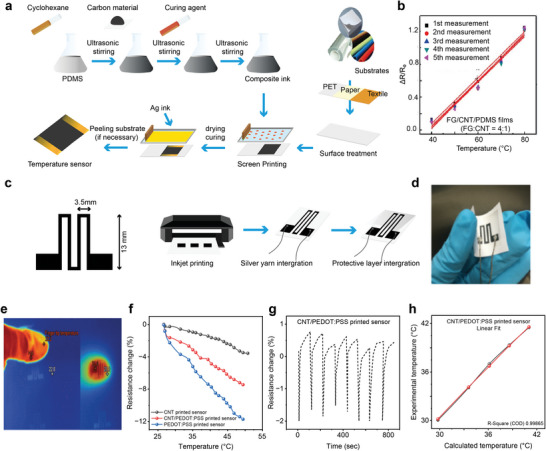
Printed textile temperature sensors a) The preparation process of temperature‐sensitive ink and sensor b) Average resistance change of 16 wt.% FG/CNT/PDMS films with FG/CNT  =  4:1 as a function of temperature. Reproduced with permission.^[^ ^165^ ^]^ Copyright 2019, Springer. c) Layout and printing of the sensor design, d) Digital image of the printed sensor, e) Thermal camera image of experimental configuration, f) Resistance change as a function of temperature for printed temperature sensors. g) Thermal response graphs of the CNT/PEDOT:PSS printed sensors when the finger touches and withdraws from the sensor, and h) Dependence of experimental data of CNT/PEDOT:PSS printed sensors with calculated data from Steinhart–Hart formula. Reproduced with permission.^[^ ^295^ ^]^ Copyright 2021, IEEE.

Wu et al.^[^ ^165^ ^]^ reported the fabrication and characterization of a flexible temperature sensor based on flake graphite (FG)/carbon nanotube (CNT)/polydimethylsiloxane (PDMS) composite‐ screen printed on PET substrate. The sensor shows high‐temperature sensitivity and good linearity. It was reported that the TCR value of the FG/CNT/PDMS films can be manipulated by the mass ratio of FG to CNT. At a mass ratio of FG to CNT is 4:1, the TCR was almost reproducible and maintained at the same level of 0.028 K^−1^ for repeated thermal cycles, indicating its potential for the flexible temperature sensor, **Figure** **10**a,b.

Some of the studies also report the exploitation of inkjet printing of conductive materials to produce temperature sensors in various substrates, especially in PEN,^[^ ^284^ ^]^ PU,^[^ ^296^ ^]^ PI,^[^ ^294^ ^]^ PET,^[^ ^201^ ^]^ rubber^[^ ^263^ ^]^ and paper.^[^ ^297^, ^298^, ^299^ ^]^ Kuzubasoglu et al.^[^ ^295^ ^]^ developed a temperature sensor based on carbon nanotube (CNT) and PEDOT: PSS‐based ink to inkjet print onto the adhesive polyamide‐based taffeta fabric, Figure 10c–h. Stable and uniform dispersions of CNT and PEDOT:PSS with Triton X‐100 were formulated using three different formulations: CNT‐based ink, PEDOT: PSS‐based ink, and CNT/PEDOT:PSS composite ink. Produced sensors exhibited NTC behavior and sensitivity of 0.15%/°C for CNT, 0.41%/°C for PEDOT:PSS, and 0.31%/°C for CNT/PEDOT:PSS for temperature varying from room temperature to 50°C. CNT/PEDOT:PSS composite ink printed sensor achieved better‐sensing repeatability and demonstrated higher bending stability with a resistance change of 0.3% up to 1000 cycles.^[^ ^295^ ^]^ Reported inkjet‐printed temperature sensors that can measure a wide range of temperatures are mostly composed of Ag‐based ink.^[^ ^201^, ^294^ ^]^ However, a unique combination of 3D printing and inkjet printing was also reported to realize a low‐cost, fully integrated wireless sensor node for large‐area monitoring applications for sensing temperature, humidity, and H_2_S levels.^[^ ^300^ ^]^

#### 5.2.4 Printed Textile Humidity Sensor

Similar to temperature, moisture is another critical factor for both the environment and living creatures. Humidity sensing is realized through the interaction between moisture and the sensing materials, such as electrolytes, semiconductor ceramics, polymers, and so forth.^[^ ^301^ ^]^ Though there are various fabrication techniques for realizing humidity sensors, printed humidity sensors offer considerably lower costs and increased capabilities for large‐scale production.^[^ ^302^ ^]^ Various printing technologies have been exploited for the fabrication of humidity sensor on several substrates, e.g. patterning on PI,^[^ ^303^ ^]^ screen printing on KAPTON,^[^ ^304^ ^]^ glass,^[^ ^305^ ^]^ PI,^[^ ^306^, ^307^ ^]^ PET,^[^ ^308^, ^309^ ^]^ gravure printing on PET^[^ ^180^, ^310^ ^]^ and PI,^[^ ^311^, ^312^ ^]^ screen and gravure combined on PET,^[^ ^181^, ^313^ ^]^ inkjet printing on PET,^[^ ^314^, ^315^, ^316^, ^317^ ^]^ PI,^[^ ^318^ ^]^ paper^[^ ^319^ ^]^ and on KAPTON,^[^ ^320^ ^]^ photolithography on LiNbO_3,_^[^ ^321^ ^]^ PI and PES^[^ ^322^ ^]^ or piezoelectric^[^ ^323^ ^]^ substrate, etc. **Table** **7** summarizes the printed textile‐based humidity sensors.

**Table 7:** Summary of printed textile humidity sensor.

Method	Substrate	Mechanism	Composed of	Humidity range	Sensitivity	Performance	Reference
Screen printing	Poly‐cotton (2013)		Ag paste with Nafion (sulfonated tetrafluoroethylene‐based fluoropolymer copolymer)	30% – 90% at temperature range of 15 °C −35 °C			[324]
	Polyester (2019)	Capacitive	Ag paste print followed by a passivation layer of polydimethylsiloxane (PDMS) and CaCl_2_	30‐ 95%	2.7‐fold greater than conventional polyimide sensor		[166]
Inkjet printing	Textile (2012)		Silver nanoparticles (Ag) ink	5–95%			[202]
	Polyester sheet (2013)		PANI	20%−100%			[325]

**Figure 11:**
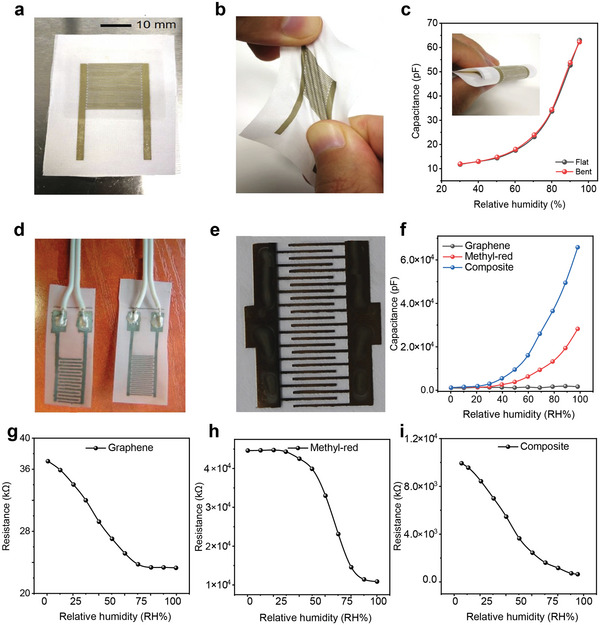
Printed textile humidity sensors a) Photograph of the sensor printed on polyester textiles b) Humidity sensor stretched along the bias c) The output of the sensor in flat and bent (radius: 5 mm) states, the inset presents a photograph of a bent sensor. Reproduced with permission.^[^ ^166^ ^]^ Copyright 2019, Elsevier. d) The humidity sensor electrodes printed on textile with Ag nanoparticles ink: sensors of left pattern–400µm × 400µm and of right pattern– 250µm × 250µm. Reproduced with permission.^[^ ^202^ ^]^ Copyright 2012, Elsevier. e) The inkjet printed sensor's electrodes with silver ink and zoom image of the area set off by a red solid line with 200 µm track width of silver electrode, f) Capacitance versus relative humidity (% RH) characteristics curves of the graphene/methyl‐red composite, methyle‐red only, and graphene only based humidity sensors measured in the humidity chamber at 1 kHz frequency g) Resistance versus relative humidity (% RH) in the graphene film, h) Resistance versus relative humidity (% RH) in the methyle‐red film, and i) Composite graphene/methyl‐red based humidity sensor. Reproduced with permission.^[^ ^315^ ^]^ Copyright 2016, Elsevier.

Screen printing technology was mostly utilized for the fabrication of humidity sensors. Kutzner et al.^[^ ^324^ ^]^ reported screen printing of Ag‐paste to fabricate humidity sensors on Polycotton fabric. It was tested between 30%‐ 90% RH at varying temperatures in the range of 15 °C and 35 °C. The complex sensor signal was measured using a gain/phase analyzer in a frequency range between 10Hz and 100kHz. It indicated a non‐linear dependency on humidity for different measurement frequencies. Komazaki et al.^[^ ^166^ ^]^ reported capacitive humidity sensor made of screen printing technology. A comb‐shaped electrode was printed with conductive silver paste followed by a PDMS passivation layer printed on the back side of the textile, **Figure** **11**a–c. The precursor emulsion of the PDMS‐CaCl_2_ micro composite was then printed on the front side of the textile. The sensor was tested over a humidity range of 30‐ 95%; the permittivity increased by 10.2% from 30 to 60%RH corresponding to a sensitivity that is 2.7‐fold greater than that of a conventional polyimide humidity sensor.

A number of researchers also employed gravure printing technology for the fabrication of humidity sensors.^[^ ^180^, ^310^, ^311^, ^312^ ^]^ Both gravure and screen printing were employed to fabricate the humidity sensors in few studies,^[^ ^304^, ^313^ ^]^ however they were not reported on textiles.

A few research groups also reported inkjet printing for humidity sensor fabrication. Weremczuk et al.^[^ ^202^ ^]^ presented a humidity sensor inkjet‐printed on textile using the ink‐jet printing technology, Figure 11d. In a controlled environment of 25 °C and 5–95% of RH, the measured impedance modulus versus humidity showed a non‐linear distribution over the range from 40 to 95% of RH. Ali et al.^[^ ^315^ ^]^ reported an Ag‐printed interdigitated electrode on a flexible PET substrate all‐printed humidity sensor based on graphene/methyl‐red composite with high sensitivity, Figure 11e–i. The sensor electrical resistance inversely varies from 11 MΩ to 0.4 MΩ against the RH content from 5% to 95%. The sensor also exhibited 96.36% and 2 869 500% resistive and capacitive sensitivity respectively against humidity. The response and recovery time of the sensor were 0.251 s and 0.35 s, respectively. Kulkarni et al.^[^ ^325^ ^]^ synthesized a conducting polyaniline‐based ink for printing an interdigitated (IDT) pattern using an inkjet printer on a polyester substrate to prepare a humidity sensor. They reported their sensor response in the humidity atmospheres ranging between 20% and 100% RH.

#### 5.2.5 Printed Textile Electrodes for Electrocardiogram

Biosensors are analytical devices that can convert biological responses into electrical signals. The term “‘biosensor”’ was coined by Cammann, and its definition was introduced by IUPAC.^[^ ^326^ ^]^ Human activities such as brain activity, heart beating, muscle, and eye movement, etc. have been widely studied for medical diagnosis^[^ ^327^ ^]^ as well as by researchers from diverse fields including neuroscience and engineering.^[^ ^328^ ^]^ Cardiovascular disease is one of the leading causes of death all over the world; however, the mortality rate could be significantly reduced simply if an electrocardiogram (ECG) test had been performed regularly on the individuals.^[^ ^329^ ^]^ Electrocardiogram is a time‐domain representation of body surface potentials, obtained by placing electrodes, originating from the heart. It is a very popular bio‐signal not only due to its distinct signals from specific regions of the cardiac system but also because the acquirement of this signal from the patient is completely free of any side effects, unlike other diagnostic methods such as X‐rays.^[^ ^330^ ^]^ ECG reflects the electric activity of the heart, usually used to determine Heart rate variability (HRV) which is important data for various medical fields.^[^ ^331^ ^]^ Therefore, recently the monitoring of electrocardiogram, and ECG has become an important tool for both clinical diagnosis and home health care, especially for long‐term monitoring.^[^ ^332^ ^]^ The conventional monitoring of ECG or EEG usually employs standard wet silver/silver chloride (Ag/AgCl) electrode which usually requires conductive gel and prior skin preparation.^[^ ^332^ ^]^ Though they have good signal performance in short periods of time and are most commonly used in hospital environments; their short shelf life, skin irritation (i.e., itchiness, reddening, swelling even allergies), and discomfort for patients limit their usage for long‐term monitoring. In addition to this, the conductivity of electrolytic gel also decreases gradually since the material gets dried, subsequently degrading the data accuracy.^[^ ^333^ ^]^ In comparison to conventional Ag/AgCl rigid metal electrodes, textile electrodes are soft, flexible, and breathable, which allows the wearer comfort more than conventional metal plate electrodes in long‐term monitoring. These can also easily be integrated into garments by weaving, knitting, or sewing, which requires no adhesive to be attached to the body, so are skin‐friendly (no skin irritation or discomfort) and environmentally friendly (electrodes are reusable). Considering these advantages, many researchers have used textile electrodes in the development of wearable ECG systems.^[^ ^334^ ^]^ Though several fabrication technologies have been employed for the fabrication of ECG biosensors, such as knitting of conductive thread,^[^ ^334^ ^]^ coating,^[^ ^335^ ^]^ embroidery^[^ ^246^, ^336^ ^]^ 3D‐printing^[^ ^337^ ^]^ or MEMS process,^[^ ^338^ ^]^ due to the scope of this review limited to printed e‐textiles, we will confine our discussion only on printed ECG sensors. **Table** **8** summarizes the reported textile‐printed electrodes employed as ECG biosensors for electrocardiogram monitoring.

**Table 8:** Summary of printed textile ECG electrodes.

Printing method	Substrate (Reporting year)	Composed of	Form/place of operation	Signal‐to‐noise ratio SNR (dB)/ performance	Reference
Brush paint	Woven and knit(2019)	Silver	Cycling suit		[339]
	Knitted Textile (2015)	PEDOT: PSS	Wristband	SNR 16.3	[340]
Screen printing	Polyester/Nylon nonwoven (2008)	Ag/AgCl ink	Chest		[167]
	Fabric (2010)	Ag paste	Band‐aid		[168]
	PET foil (2013)	Ag/AgCl‐based ink		Stable, very small potential drift (<3 mV/30 min)	[341]
	Escalade (Cotton/polyester / Lycra), 3 × 1 twill woven (2014)	Ag paste/PU paste	Chest band		[170]
	Lagonda (Cotton/polyester/lycra woven (2015)	Ag paste/carbon‐loaded rubber	Chest band		[342]
	Polypropylene nonwoven (2015)	Ag/AgCl conductive inks	Chest	SNR 28.68 (dry), 26.70 (wet)	[343]
	Cotton Woven (2016)	PEDOT: PSS			[344]
	Woven (2017)	Ag paste/conductive rubber	Body vest		[345]
	Textile substrate (2017)	PEDOT:PSS conductive organic polymer			[346]
	Knit t‐shirt (2017)	PEDOT: PSS	Chest	SNR 15.42 (Dry), 29.59 (Wet)	[82]
	Cotton (2018)	PEDOT: PSS			[347]
	Cotton knit (2018)	PEDOT: PSS	Forearms	Before Washing SNR 24.63, after 11.8333 (7.4 wt.%), 33.0505 and 7.6069 (12.8 wt.%) respectively	[83]
	Cotton/lycra knit(2018)	PEDOT: PSS		Before Washing SNR 17.8022, after 11.5040 (6.3 wt.%), 27.7690 and 15.6060 (11.9 wt.%) respectively	[83]
	Ag‐plated cotton	PEDOT:PSS		Before washing 34.7203 and after washing 33.1449	[83]
	Textile (2018)	Ag conductive ink			[348]
	PET (2018)	Ag ink print followed by MWCNT/ PDMS composite polymer bar coat		correlation coefficient of 0.95	[349]
	Textile (2018)	Silver ink and PEDOT: PSS ink	Chest	SNR 21	[350]
	Cotton (2019)	Graphene ink/ Carboxymethyl Cellulose (CMC)	Wrist		[351]
	Woven PU‐coated fabric (2019)	MWCNT with PU binder	Chest		[352]
	Cotton knit fabric (2020)	Silver ink		33.10 (cotton), 33.52 dB (Ag/AgCl electrodes)	[353]
	Polyester knit fabric (2020)	Silver ink		30.17 (polyester), 33.52 dB (Ag/AgCl electrodes)	[353]
	Thermoplastic polyurethane (TPU) (2020)	Ag, Ag/PEDOT:PSS, Ag/AgCl, Ag/AgCl/PEDOT:PSS, C, C/AgCl, and PEDOT:PSS	Anterior wrist on opposite arms	All electrodes show *r* ^2^ > 0.95, indicating a nearly perfect overlap of the gel and dry electrodes	[354]
	PET (2020)	Laser‐induced graphene (LIG), AgCl, carbon inks	Wrist		[355]
	Cotton (2020)	Graphene ink/ Ethyl cellulose (EC)	Wrist		[356]
Inkjet printing	PET (2015)	Ni particle on activator			[357]
	Hosiery (Pantyhose) (2017)	PEDOT: PSS	Wrist	SNR 12.93 ± 0.80 (dry) and 13.75 ± 0.26 (gel)	[358]
	Cotton (2017)	Reduced graphene oxide (rGO)	Fingertip	SNR 22.3	[147]
	Woven (2019)	Reactive silver	Elbow	SNR 18	[359]

The performance of biosensors is determined in terms of various parameters. The most common parameter is the signal‐to‐noise (SNR) ratio. This is useful data to measure the quality of recordings, as well as helpful for evaluating a process flow during data analysis, tuning its variable parameters, and diagnosing problematic areas that require additional improvement or refinement. SNR is typically defined as a ratio of signal energy to noise energy.^[^ ^360^ ^]^ For any biosensor, A higher signal‐to‐noise ratio (SNR) leads to a less complicated algorithm in the beat detection step and thus increases in accuracy.^[^ ^361^ ^]^

**Figure 12:**
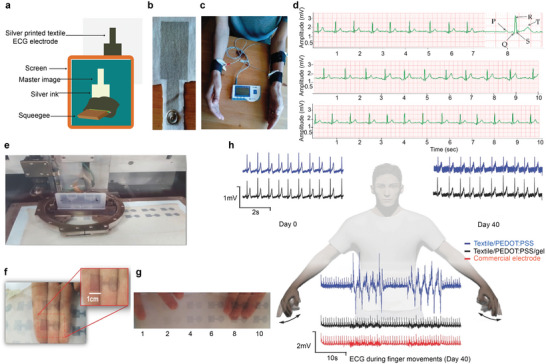
Printed textile electrodes for ECG measurement a) Block diagram for the screen printing process, b) Front view of the developed electrode; c) Component placement setup for ECG measurement, d) ECG signals collected using: (top) silver printed cotton; (middle) silver printed polyester fabric; and (bottom) Ag/AgCl gel electrode. Reproduced with permission.^[^ ^353^ ^]^ Copyright 2020, The Authors. e) Photograph of the inkjet printing process, f) Photograph of printed electrodes on a commercial textile with a zoom on an individual electrode, g) Photograph of electrodes with a different number of printed layers (1, 2, 4, 6, 8, 10) h) ECG data acquired under different conditions: static recoding at t0 in the top left corner, i) Static recording at t0 + 40 d in the top right corner, dynamic recording under repeated hand motion at t0 + 40 d in the bottom center. Reproduced with permission.^[^ ^358^ ^]^ Copyright 2017, WILEY‐VCH.

Sinha et al.^[^ ^82^ ^]^ utilized screen‐printing of PEDOT:PSS electrodes on a t‐shirt to present a platform for monitoring the cardiovascular activity of an athlete both during sedentary conditions and during exercise. PEDOT:PSS is known to be a mixed conductor to functions as both ionic and electronic conductors, in which PEDOT is responsible for electronic conductivity, and PSS contributes to ionic conductivity, both of which are necessary for transduction of the ECG signal. Without using any hydrogel or an adhesive around the electrodes, the ECG signals were recorded in dry skin conditions. The signal amplifies when the skin transpires water vapor or by applying a common lotion on the skin (SNR were 15.42 for dry, and 29.59 in wet condition). The PEDOT:PSS wires connected to PEDOT:PSS electrodes have been shown to record ECG signals comparable to Ag/AgCl connected to copper wires. Ankhili et al.^[^ ^83^ ^]^ reported a comparative study of pure cotton and cotton/Lycra knitted fabric to make flexible textile electrodes by screen‐printing of PEDOT:PSS conducting polymer. Ag‐plated electrodes were also used to compare their performance with developed PEDOT:PSS textile electrodes. The pure cotton knit textile electrodes exhibited better SNR than the cotton‐lycra knit textiles; highest SNR of 33.0505 (before washing) and 7.6069 (after washing) in comparison to 27.7690 (before washing) and 15.6060 (after washing). However, the highest SNR was reported for Ag‐plated cotton; 34.7203 (before washing) and 33.1449 (after washing). Ag ink was also used as conductive material for fabricating textile‐based ECG electrodes,^[^ ^353^ ^]^ **Figure** **12**a–d. The ECG signals collected using textile electrodes were comparable to the standard Ag/AgCl electrodes with an SNR of 33.10, 30.17, and 33.52 dB for signals collected from cotton, polyester, and Ag/AgCl electrodes, respectively. Zalar et al.^[^ ^354^ ^]^ compared the electrical performance of seven screen‐printed dry electrodes (Ag, Ag/PEDOT:PSS, Ag/AgCl, Ag/AgCl/PEDOT:PSS, C, C/AgCl, and PEDOT: PSS) with two commercially available gel electrodes (3M RedDot 50 and Philips NeoLead) on thermoplastic polyurethane substrates. They reported the performance of the electrodes in terms of correlation coefficient. All the screen‐printed dry electrodes showed an *r* ^2^ > 0.95, indicating nearly perfect overlap with the commercial gel electrodes, i.e., the collection of ECG signals with a quality equal to that of gel electrodes.

Inkjet printing is by far the fastest‐growing sub‐segment in textile printing. Due to providing a number of advantages over traditional printing methods, e.g., screen printing, roller printing, and transfer printing, such as versatility, great resolution, customizing flexibility, reduced downtime and sampling cost, lower waste output, and usage of water and chemicals, it has been growing rapidly.^[^ ^362^ ^]^ Therefore, inkjet printing was employed for fabricating ECG electrodes on various substrates including paper,^[^ ^363^ ^]^ tattoo paper,^[^ ^364^ ^]^ PI substrate,^[^ ^365^ ^]^ etc. Bihar et al.,^[^ ^358^ ^]^ inkjet printed PEDOT:PSS on a commercial stretchable textile to make electrodes, Figure 12e–h. Skin contact was improved by the addition of a cholinium lactate‐based inkjet printed ionic liquid gel on the textile. The gel‐assisted electrodes made low‐impedance contacts with the skin and the accuracy of data was comparable with the commercial wet Ag/AgCl electrodes. However, the common problem associated with inkjet printing of conductive inks on textiles is the difficulty of printing a continuous conductive path on a rough and porous textile surface. To overcome this issue, we reported for the first time the inkjet printing of an organic nanoparticle‐based surface pre‐treatment onto textiles to enable all inkjet‐printed graphene e‐textiles.^[^ ^147^ ^]^ The functionalized organic nanoparticles presented a hydrophobic breathable coating on textiles followed by an inkjet printing of rGO to form a continuous conductive electrical path. We demonstrated the inkjet‐printed e‐textile as a biosensor to monitor the heart rate with a high SNR of 22.3.

#### 5.2.6 Printed textile electrodes for Electroencephalography

Electroencephalography (EEG) is a common technique to record the electrical activity of the brain. In sleep studies, it is a compulsory part of polysomnography (PSG) to detect sleep stages and cortical arousals.^[^ ^366^, ^367^ ^]^ This is a non‐invasive measurement^[^ ^368^ ^]^ with negligible health risk and minimal restriction on the users’ age. It is regularly monitored along with other neuro imaging techniques such as magnetic resonance imaging (MRI) in the clinic to diagnose neurological disorders such as epilepsy, sleep disorders, and attention deficit hyperactivity disorder (ADHD) as well as to evaluate patients with a history of depression and other psychiatric disorders.^[^ ^328^, ^369^ ^]^ EEG detects both mind‐wandering and mental efforts.^[^ ^370^, ^371^ ^]^ Electrode is the most critical component in any device for monitoring biopotentials; where the transduction of potential signals in the tissue to the solid state conductor takes place. Skin surface electrode is especially important in the case of EEG, where the nature of the interface between the recording electrode and the scalp can overwhelmingly influence signal quality and sensitivity. A clinical scalp‐EEG setup typically requires a standard 10–20 electrode placement set in which 21‐cup electrodes, commonly made of tin, gold, silver, silver‐silver chloride, or platinum, are attached to the scalp at precise locations to record the potential differences. However, several factors make the application of conventional electrodes challenging.^[^ ^372^ ^]^ It requires a long setup time, and special skills to find correct positions and to create good electrode‐skin contact, especially through the hair. Also, conventional EEG electrodes are not usually MRI‐compatible. They can cause artifacts in MRI images and the alternating magnetic field can cause tissue heating or electrode (wire) movement. Thirdly, solid metal cup electrodes do not conform properly to the skin, which causes motion artifacts in the EEG signal.^[^ ^373^ ^]^ Development of an effective EEG electrode requires consideration of several factors, including impedance, susceptibility to recording artifacts, long‐term stability, safe skin contact, and several practical considerations such as size, weight, simplicity of application, and cost.^[^ ^374^ ^]^ Textile‐based EEG electrodes were reported by several research groups on different substrates utilizing different conductive materials; pin shaped carbon fiber electrode,^[^ ^375^ ^]^ Ag‐thread, Ag‐plated nylon, spandex, PP knitted textiles,^[^ ^376^, ^377^ ^]^ Ni/Cu coated on polyester^[^ ^378^ ^]^ or conductive polymer treated fabric,^[^ ^379^ ^]^ PANI coated on PU foam,^[^ ^380^ ^]^ electroless copper plating on polyester fabrics^[^ ^381^ ^]^ or EEG electrodes placed in textile hats,^[^ ^382^ ^]^ etc. However, printed textiles have not been widely investigated yet. **Table** **9** summarizes the printed textile EEG electrodes for monitoring brain activity.

**Table 9:** Summary of printed textile electrodes for EEG.

Printing method	Substrate (Reporting year)	Composed of	Type	Performance	Reference
Screen print	Textiles (2015)	Ag paste, conductive rubber	Headband	Accuracy of 90 (±9) %	[169]
	Woven fabric (2017)	Ag paste, conductive rubber	Headband	Accuracy > 90%	[383]
	Cotton Woven (2022)	Graphene	Forehead	Correlation coefficient > 0.998	[151]
Jet printing	Polyurethane nonwoven (2018)	Composite ink of Ag and flouro‐elastomer in methyl ethyl ketone (MEK)	The hairless region behind the ears		[203]

**Figure 13:**
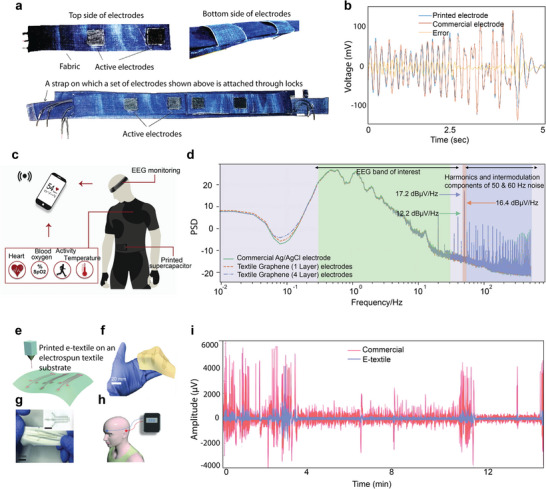
Printed textile electrodes for EEG measurement. a) Top and bottom side of printed electrodes (Top) and the overview of the headband (Bottom) b) EEG electrode comparison results. Reproduced with permission.^[^ ^383^ ^]^ Copyright 2017, Elsevier. c) Schematic of placement of EEG headband d) Power spectral density of recorded signal from graphene textiles electrodes against commercial Ag/AgCl electrodes, highlighting the typical frequency band of interest in EEG studies shaded in green. The 50 Hz component and its contribution for each of the electrode types are shaded in red. Also shown are higher frequency harmonics and intermodulation components from 50 to 60 Hz noise shaded in dark blue. Reproduced with permission.^[^ ^151^ ^]^ Copyright 2022, The Authors. e) The conceptual drawing of jet‐printing nanocomposite inks onto a textile substrate f) A sheet of e‐textile with printed conductive serpentine traces. g) Top view photographs of the two‐layered e‐textile seen from the serpentine traces sides. The main panels and the insets (scale: 10 mm) show stretched and non‐stretched states, respectively. h) Schematic illustration of EEG recording from behind the ear i) Raw data collected from a commercial gel EEG electrode behind the left ear, simultaneously with an e‐textile electrode behind the right ear (blue). Larger spikes in noise are observed for the commercial sensor due to less mechanical conformation with the skin. Reproduced with permission.^[^ ^203^ ^]^ Copyright 2018, WILEY‐VCH.

Wei et al.,^[^ ^383^ ^]^ screen‐printed Ag‐paste with conductive rubber on woven textiles to realize a real‐time emotion detection system based on EEG measurement. They used an emotion detection headband coupled with printed signal acquisition electrodes and open source signal processing software (OpenViBE), **Figure** **13**a,b. The subject‐dependent accuracy, using improved locations, increased to 91.75% from 86.83%. 75% of the participants achieved a classification accuracy >90%, compared with only 16% of participants before improving the electrode arrangement. In our previous work,^[^ ^151^ ^]^ we demonstrated screen‐printed graphene‐based textile electrodes to record EEG by simulating the EEG part of a PSG using two electrodes and a gelatine head phantom. The correlation coefficient between filtered data collected from graphene‐based electrodes over a 20‐min record was >0.998, indicating the very similar performance of our electrodes with conventional rigid Ag/AgCl electrodes, Figure 13c,d. La et al.^[^ ^203^ ^]^ reported two‐layered e‐textile patches with high mechanical durability and electrical performance, fabricated by jet‐printing. Ag‐powder/fluoropolymer‐based nanocomposite ink on both sides of the porous textile substrate. They demonstrated their electrode to record true brain signals of the participants while opening and closing their eyes, Figure 13e–i.

#### 5.2.7 Printed Textile Electrode for Electromyography

Electromyography (EMG) is a technique for recording and evaluating electrophysiological signals related to muscle activity (also referred to as the myoelectric signal). This is a fundamental method for understanding the muscle activity of the human body under normal and pathological conditions.^[^ ^384^ ^]^ Surface EMG is another non‐invasive, painless, and easy technique to assess the myoelectric signal, utilized in various applications including clinical diagnosis of neuromuscular disorders, the study of muscle fatigue, and control of prosthetics, etc.^[^ ^385^ ^]^ This is important especially for measuring the electrical activity of muscles without function due to injury or disease as well as active prostheses movement control helping amputees to regain independent and unrestricted life.^[^ ^386^ ^]^ EMG is also important to infer motion intention and therefore could be used to control devices such as exoskeletons, biofeedback systems, or assistive tools.^[^ ^387^ ^]^

Textiles were employed as EMG electrodes fabricated through a number of methods. Silver array mechanically attached to fabrics,^[^ ^387^, ^388^ ^]^ conductive thread sewn/embroidered into fabrics,^[^ ^389^, ^390^, ^391^ ^]^ stainless steel/cotton yarn sewn/embroidered into fabrics,^[^ ^392^ ^]^ nickel plated copper conductive woven fabric,^[^ ^393^ ^]^ PPy coated on nonwoven sheets,^[^ ^394^, ^395^, ^396^ ^]^ PPy coated on woven fabrics and yarns, coated yarn then knit,^[^ ^397^ ^]^ PEDOT:PSS selectively coated on knit fabric,^[^ ^398^ ^]^ Ag coated on PA yarn to prepare conductive woven,^[^ ^399^, ^400^, ^401^, ^402^, ^403^ ^]^ embroidered on woven,^[^ ^404^ ^]^ conductive knit fabric,^[^ ^405^, ^406^, ^407^ ^]^ SS and polyester stable fiber yarn conductive knit^[^ ^407^ ^]^ multilayer fabric^[^ ^408^ ^]^ are among the reported textile EMG electrodes. **Table** **10** provides a brief overview of printed textiles for EMG measurement.

**Table 10:** Summary of printed textile electrodes for EMG.

Printing method	Substrate	Composed of	Form/place of operation	Signal‐to‐noise ratio SNR (dB)/Performance	Reference
Stencil printing	Nylon/PU tricot knit (2017)	Flouro‐elastomer/silver composite ink	Arm		[410]
Screen printing	Woven Textile (2012)	Conductive ink	Forearm		[411]
	Escalade (Cotton/polyester / Lycra), 3 × 1 twill woven (2014)	Ag paste/PU paste	Armband		[170]
	Woven (2014)	Ag paste, PU paste	Headband		[412]
	Cotton (2019)	PEDOT: PSS	Tibialis anterior muscle		[413]
	PET (2020)	Laser‐induced graphene (LIG), AgCl, carbon inks			[355]
	Polyester‐cotton fabric (2021)	Ag‐polymer ink	Forearm	accuracy of ≈93%	[409]
	Textile and a silver plated lycra fabric (CCSM) (2022)	Ag, PEDOT:PSS			[414]
Jet printing	Polyurethane nonwoven (2018)	Composite of Ag and flouro‐elastomer in methyl ethyl ketone (MEK)	Several body part		[203]

**Figure 14:**
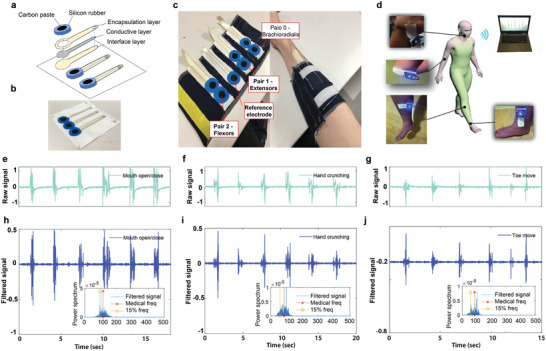
Printed textile electrodes for EMG measurement. a. Exploded view of printed electrodes. b. Complete screen‐printed electrode c. Final measurement device. Reproduced with permission.^[^ ^409^ ^]^ Copyright 2021, The Authors. d) Schematic illustration of sEMG measurements from various parts of the human body. e) The raw and h) filtered sEMG signals from mouth open/close episodes measured from the submental space. f) The raw and i) Filtered sEMG signals from hand crunching episodes measured from the elbow. g) The raw and j) Filtered sEMG signals from toe lifting episodes measured from the ankle. Insets are power spectrums obtained by Fourier transforms of each data. Reproduced with permission.^[^ ^203^ ^]^ Copyright 2018, WILEY‐VCH.

Tao et al.^[^ ^411^ ^]^ screen printed conductive ink on woven textiles to demonstrate the real‐time performance of as‐printed electrodes in EMG pattern‐recognition‐based prosthesis control. Their results tested on seven participants exhibited an accuracy of 91.81% for ten different movements, compared to 96.54% for six basic movements. Pani et al.^[^ ^413^ ^]^ demonstrated the performance of polymer‐based screen‐printed textile electrodes for sEMG signal detection. They deposited PEDOT:PSS onto cotton fabric to form the electrode. While compared with the conventional electrodes, the *r* ^2^ value was >97% for all measurement conditions. Court et al.^[^ ^409^ ^]^ reported a screen‐printing process to deposit four functional layers in turn, on a polyester cotton fabric to realize an EMG electrode, **Figure** **14**a–d. An interface layer was printed to smoothen the surface of the fabric and improve printability for the subsequent conductive layer. The conductive layer consists of a silver polymer ink for the conductive tracks, followed by encapsulation using the same ink as the interface layer. The final layer was a stencil‐printed conductive carbon rubber paste covering one of the exposed conductor areas, providing a dry electrode contact to the skin. A total of five gestures were uniquely identified with an average accuracy of ≈93% when operating with a switching delay of 150 ms or greater. As described in the previous section the work of La et al.,^[^ ^203^ ^]^ reported a jet‐printed two‐layered e‐textile patches of Ag‐powder / fluoropolymer‐based nanocomposite ink on both sides of the porous textile substrate. They controlled the thickness of the cladded layer which provided a remarkable advantage in designing electrodes for both EMG and EEG applications. A complete wireless EMG system was also demonstrated with their custom‐designed data acquisition/transmission electronics, Figure 14e–i.

#### 5.2.8 Printed Textile Electrodes for Electrooculography

Electro‐oculography (EOG) is safe, convenient, and efficient thus been the standard eye‐movement measurement technique for many decades.^[^ ^415^ ^]^ Based on the corneoretinal potential (difference in electrical charge between the cornea and the retina), with the long axis of the eye acting as a dipole, eye movements relative to the surface electrodes placed around the eye produce an electrical signal that corresponds to eye position.^[^ ^416^ ^]^ Graphene‐coated textiles were reported as EOG electrodes.^[^ ^417^ ^]^ **Table** **11** summarizes the printed textiles utilized for the acquisition of EOG.

**Table 11:** Summary of printed textile electrodes for EOG.

Printing method	Substrate (Reporting year)	Composed of	Type	Reference
Screen print	Polyester (2013)	Ag ink	Face to face	[171]
	Escalade (Cotton/polyester / Lycra), 3 × 1 twill woven (2014)	Ag paste, PU paste	Headband	[170]
	Woven (2014)	Ag paste, PU paste	Headband	[412]
	PET (2020)	Laser‐induced graphene (LIG), AgCl, carbon inks	Forehead	[355]

**Figure 15:**
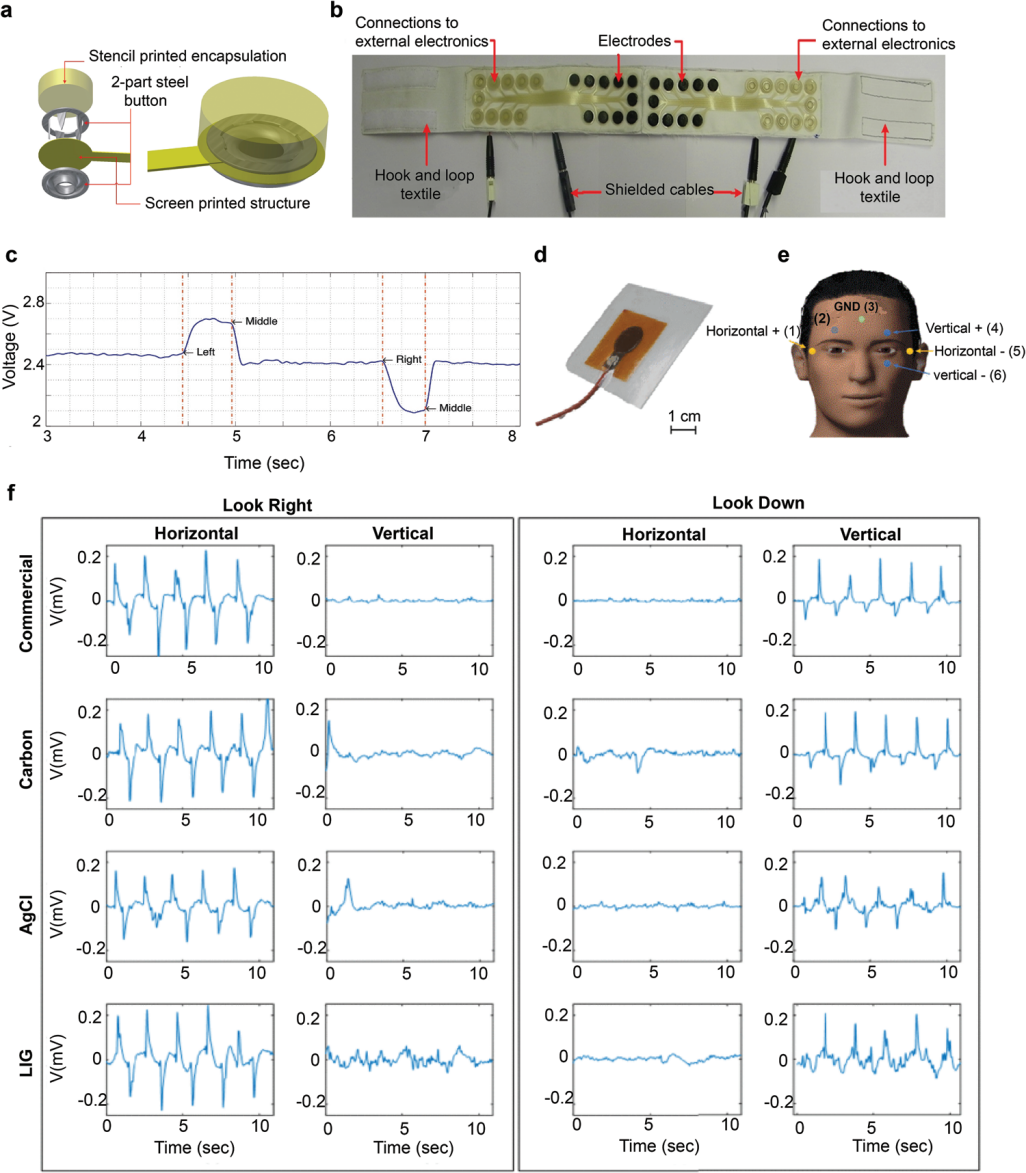
Printed textile electrodes for EOG measurement. a) Fabrication of electrode components, left: individual parts and right: completed electrode. b) Textile headband for EMG and hEOG monitoring. c) hEOG recorded with the textile printed headband. Reproduced with permission.^[^ ^170^ ^]^ Copyright 2013, Elsevier. d) Multilayer structure of the developed LIG electrode e) Positions of the electrodes and signal nomenclature. f) EOG signals for different materials and eye movements. Reproduced with permission.^[^ ^355^ ^]^ Copyright 2020, The Authors.

Myllymaa et al.^[^ ^171^ ^]^ utilized screen‐printing technology to construct the electrode set consisting of ten electroencephalography (EEG) electrodes, two electrooculography (EOG) electrodes, two ground electrodes, and two reference electrodes. Paul et al.,^[^ ^170^ ^]^ developed a screen‐printed network of electrodes and associated conductive tracks on textiles for medical applications. A polyurethane paste was screen printed on to a woven textile to create a smooth, high surface energy interface layer. A silver paste was subsequently printed on top of that to provide a conductive track followed by another PU encapsulation layer to protect the silver track from abrasion and creasing. Conductive rubber, with a thickness of 3 mm, was then stencil printed on to the terminations of these conductive tracks to form electrodes. The electrodes, used in contact with the skin, in the form of a headband, were demonstrated and evaluated for the biopotential monitoring applications of ambulatory ECG, EMG, and EOG, **Figure** **15**a–c. The same research group further reported^[^ ^412^ ^]^ a printed electrode headband, used in a facial EMG and EOG control system. The system was also used to control a mouse cursor or simulate keyboard functions. It was found that 50 Hz noise levels in the printed textile electrodes were similar to commercial disposable electroencephalography electrodes. The effect of a wearable approach on pressure variations and motion artifacts is examined. Toral et al.,^[^ ^355^ ^]^ compared three types of electrodes based on (laser‐induced graphene) LIG, silver chloride, and carbon inks during the acquisition of biopotentials including ECG, EMG, and EOG. They also developed a completely new framework for the acquisition of EOG based on a printed patch that integers 6 electrodes for the EOG acquisitions and an ad‐hoc signal processing to detect the direction and amplitude of the eye movement, Figure 15d–f. Performances of the developed electrodes were compared with commercial ones using the characteristics parameters and showed similar performance with the commercial electrodes with an improvement in the comfort of the user.

### 5.3 Printed Textile Supercapacitors

The continuous development of science and technology increases the demand for energy storage and conversion equipment enormously.^[^ ^418^ ^]^ To facilitate the wide growth of wearable electronics technology, it is necessary to provide a constant power supply that is flexible, durable, lightweight, biocompatible, and strong.^[^ ^137^, ^204^ ^]^ Currently existing energy storage devices are rigid and bulky and do not demonstrate fabric‐like properties such as hand feel, thickness, or drape. Moreover, the integration of multiple components of e‐textiles must be systematic to be a part of the garment which also needs to be flexible, light in weight, and highly functional in performance.^[^ ^172^ ^]^ Therefore, there is a strong need to develop small, flexible, and high‐performance energy storage devices for easy integration with wearable electronics.^[^ ^419^ ^]^ Supercapacitors (SCs) or ultracapacitors are promising devices for energy storage having characteristics that lie between dielectric capacitors and conventional batteries.^[^ ^420^ ^]^ Electric double‐layer capacitors (EDLCs) and pseudo‐capacitors are two main types of supercapacitors; EDLCs offer a high power density, good reversibility, and a long cycle life whereas pseudo‐capacitors have a much higher energy density and a lower cycle life than EDLCs.^[^ ^421^ ^]^ Thin and flexible supercapacitors, due to possessing features such as lightweight, high power density, and their ability to deliver under mechanical deformation conditions are gaining more consideration for wearable electronics. However, the insufficient energy density still limits their use in practical applications.^[^ ^204^ ^]^ Enormous efforts have been carried out by researchers to meet these demands for making multifunctional e‐textiles and adding value to conventional textiles.^[^ ^147^ ^]^ Research regarding the integration of supercapacitors with other flexible textile electronics thus offers the solution of powering wearable e‐textiles.^[^ ^422^ ^]^ Simple fabrication methods such as photolithography,^[^ ^423^, ^424^ ^]^ coating,^[^ ^425^, ^426^, ^427^ ^]^ painting or spraying^[^ ^428^, ^429^ ^]^ or laser scribing,^[^ ^430^, ^431^ ^]^ sputtering,^[^ ^432^, ^433^ ^]^ have been employed for the fabrication of supercapacitors. However, printing technique has also been employed for supercapacitor fabrication in various substrate such as paperboard,^[^ ^434^ ^]^ PET film,^[^ ^435^ ^]^ PEN, PDMS, etc. Printed electronics represents a paradigm shift in the manufacturing of textile‐based supercapacitors in that it provides a whole range of simple, low‐cost, time‐saving, versatile, and environmentally friendly manufacturing processes.^[^ ^436^ ^]^ **Table** **12** summarizes the printed textiles employed for supercapacitor fabrication.

**Table 12:** Summary of Printed Textile Supercapacitors.

Printing Method	Substrate (Reporting year)	Device Configuration	Device capacitance	Energy density	Power density	Capacitance retention	Flexibility	Reference
Screen Printing	Cotton and polyester fabric (2011)	Cotton and polyester screen printed with AC as electrode, polyester separator, and Li_2_SO_4_ and Na_2_SO_4_ electrolyte	Electrode 0.43 F cm^−2^ at 5 mA cm^−2^ (Na_2_SO_4_), 85–95 F g^−1^ at 1–10 mV s^−1^			92% after 10 000 cycles		[437]
	Carbon fiber knit and woven fabric (2013)	Carbon fabric screen printed with AC with solid polymer electrolyte	88 F g^−1^, 0.51 F cm^−2^ (Knitted), 66 F g^−1^, 0.19 F cm^−2^ (Woven)				80% after bending at 90°, 135°, and 180°	[172]
	Cotton, polyester, spandex, 50/50 NycO, Sigma 4 Star, Defender M14 Stretch, Nonwoven 3NP (2016)	Screen printed AC ink with ionic liquid electrolyte (1‐ethyl‐3‐methylimidazolium tetracyanoborate) and graphene foil as current collectors	20 mF cm^−2^ and 4.21 F g^−1^					[438]
	Silk fabric (2016)	Screen printed current collector and active materials layers [MnO‐coated hollow carbon microspheres, acetylene black, and binder in 7:2:1] on commercial silk fabrics and PDMS film, respectively. PDMS‐based electrode was pasted with gel electrolyte and transferred on the top of the silk fabric‐based electrode	19.23 mF cm^−2^ at 1 mA cm^−2^			84% after 2000 cycles	98.5% after 100 times bending and 96.8% after 100 times twisting	[439]
	Carbon cloth (2017)	SnO_2_ nanoparticles, CNTs, ethyl cellulose, and terpineol composite ink screen‐printed onto carbon cloth. Furnace‐calcined SnO_2_/CNT electrodes sandwiched with PVA‐H_2_SO_4_ gel electrolyte	5.61 mF cm^−2^ (flat) and 5.68 mF cm^−2^ (bent)					[440]
	Cotton Woven (2017)	Screen printing of GO, followed by electrochemical reduction to produce rGO‐cotton electrode, with PVA‐H_2_SO_4_ gel electrolyte	2.5 mF cm^−2^, 257 F g^−1^			97% after 10 000 cycles	95.6% after folding 180° for 2000 cycles	[441]
	Stretchable textiles (2018)	Fully printed Ag@PPy@MnO_2_ on Ag cathode electrode and activated carbon on Ag anode electrode with PVA‐Na_2_SO_4_ electrolyte	426.3 mF cm^−2^ (cathode), device 95.3 mF cm^−2^	0.0337 mWhcm^−2^ at 0.38 mW cm^−2^		90.8% after 5000 cycles	86.2% after 40% stretching strain	[442]
	Polyester fabric (2020)	Ag paste printed on PET, MnHCF‐MnOx/GO ink overprinted and reduced to form the electrode, PVA‐LiCl electrolyte, and paper separator	16.8 mF cm^−2^	0.5 mWhcm^−2^	0.0023 mW cm^−2^		Stable at bending to angles 60°, 90°, 180° for 100 cycles	[443]
	Cotton fabric (2022)	Graphene ink screen printed on cotton textiles with PVA‐H_2_SO_4_ gel electrolyte	3.2 mF cm^−2^	0.28 mWhcm^−2^ at 3 mW cm^−2^		95% after 10 000 cycles		[151]
Inkjet printing	Polypropylene (PP) fabric (2019)	Reactive inkjet printing of rGO layers on PP fabric as an electrode with PVA‐H_3_PO_4_ gel electrolyte to form flexible solid‐state SC	13.3 mF cm^−2^ (79.9 F g^−1^) at 0.1 mA cm^−2^	1.18 mWhcm^−2^	4.6 mW cm^−2^	Almost 100% after 5000 cycles		[444]
	carbon cloth (2019)	Nickel cobalt layered double hydroxide‐(LDH/Ag/rGO) as positive and activated carbon as negative electrode with 1 M KOH electrolyte	95 mAh g^−1^ at 0.6 A g^−1^	76 Whkg^−1^	480 Wkg^−1^	79.8% after 5000 cycles		[445]
	Bamboo fabric (2020)	MnO_2_–NiCo_2_O_4_ printed bamboo fabric as positive electrode, and rGO inkjet printed bamboo fabric as negative electrode with LiCl/PVA gel as solid‐state electrolyte	2.12 F cm^−2^ (1766 F g^−1^) at 2 mA cm^−2^	37.8 mW cm^−3^	2678.4 mW cm^−3^	92% of after 5000 cycles		[204]
	PP non‐woven textile (2021)	Reactive inkjet printing to fabricate PPy layers on textile substrates with direct freezing of inks	72.3 F g^−1^ at 0.6 A g^−1^ at −12 °C	6.12 Wh kg^−1^	139 W kg^−1^	55.4% after 2000 cycles		[446]

**Figure 16:**
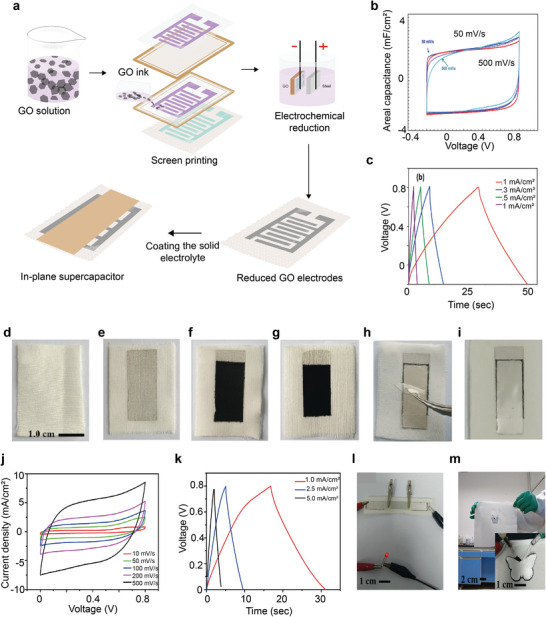
Screen printed textile supercapacitors a) Schematic representation of the printed SC fabrication process. Electrochemical characterization of the printed graphene on textile: b) CV at different scan rates and c) Charge–discharge curves at different current densities. Reproduced with Permission.^[^ ^441^ ^]^ Copyright 2017, IOP Publishing Ltd. From a silk fabric to a silk fabric‐based fully printed supercapacitor: d) Piece of pristine silk fabric, e) Silk fabric after successively screen printing of silver, f) Carbon, and g) Active materials layers, h) Removal of the PDMS support from the stacked device, i) Single textile‐supported supercapacitor. Electrochemical characterization of the supercapacitor: j) CV curves of a supercapacitor at various scan rates. k) Galvanostatic charge–discharge curves over a potential window from 0 to 0.8 V at different current densities, l) Digital photograph of a red LED powered by three supercapacitors connected in series, and m) Digital photograph of a specially designed supercapacitor with a butterfly pattern. Reproduced with permission.^[^ ^439^ ^]^ Copyright 2016, American Chemical Society.

Jost et al.^[^ ^437^ ^]^ screen printed porous carbon materials on woven cotton and polyester fabric for fabricating flexible and lightweight electrodes for supercapacitor fabrication. Utilizing a polyester separator and Li_2_SO_4_ and Na_2_SO_4_ electrolyte, they reported a high gravimetric capacitance of 85–95 F g^−1^ and areal capacitance of ≈0.43 F cm^−2^ at a scan rate of 1–10 mV s^−1^. In our previous work,^[^ ^441^ ^]^ we printed graphene oxide (GO) ink on cotton woven textiles, followed by electrochemical reduction to produce rGO‐cotton electrode, **Figure** **16**a–c. Using a PVA‐H_2_SO_4_ gel electrolyte, our device achieved an areal capacitance of 2.5 mF cm^−2^ and a high specific capacitance of 257 Fg^−1^ which retained its capacitance up to 97% even after 10 000 charge–discharge cycles and up to 95.6% after folding at 180° for 2000 cycles. In our other work,^[^ ^151^ ^]^ we utilized the screen printing process to demonstrate a graphene‐based multifunctional e‐textile platform. Produced e‐textiles were extremely flexible, conformal, and able to perform as a piezoresistive sensor for activity monitoring. The printed in‐plane supercapacitor provided an aerial capacitance of ∼3.2 mFcm^−2^ (stability ≈10 000 cycles). It was also used to record brain activity (EEG) and find comparable to conventional rigid electrodes. One of the high‐performing supercapacitors fabricated by screen printing was reported by Liu et al.^[^ ^442^ ^]^ Ag current collector, Ag@PPy@MnO_2_ composites as a cathode electrode, activated carbon as anode electrode, and gel electrolyte were printed onto a stretchable textile. The cathode electrode exhibited a high areal capacitance (426.3 mFcm^−2^ at 0.5 mAcm^−2^) and superior cycling ability (98.7% capacitance retention even after 10 000 cycles). The all‐printed stretchable asymmetric supercapacitor (ASCs) exhibited an areal capacitance of 95.3 mF cm^−2^, energy density of 0.0337 mWh cm^−2,^ and 86.2% retention after 40% stretching strain. Zhang et al.,^[^ ^439^ ^]^ involved both screen printing and transfer printing to construct all‐solid‐state supercapacitors on a single silk fabric, Figure 16d–m. The system exhibited a high areal capacitance of 19.23 mF cm^−2^ at a current density of 1 mAcm^−2^ and excellent cycling stability with capacitance retention of 84% after 2000 charging/discharging cycles with stable performance and structures after 100 times bending and twisting.

**Figure 17:**
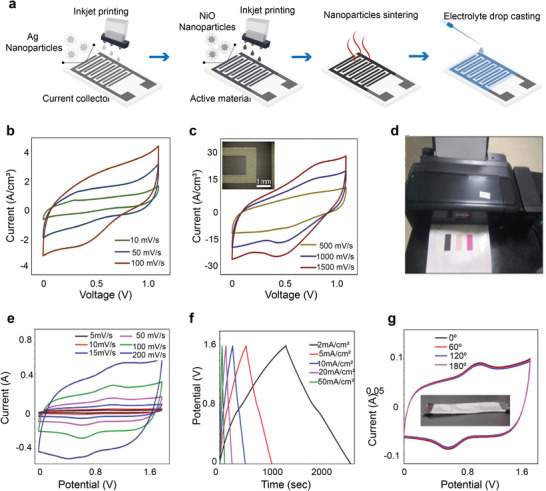
Inkjet printed textile supercapacitors a) Fabrication process of Inkjet Printed NiO supercapacitors. b, c) CV curves of supercapacitors printed on PET with adhesion promoting layer. The devices were scanned at voltage a window from 0 to 1 V at scan rates from 10 to 1500 mV s^−1^. The interdigitated electrodes and interelectrode gaps of the inset images are 1000 and 500 µm wide respectively. Reproduced with permission.^[^ ^447^ ^]^ Copyright 2020, Elsevier Ltd. d) Photograph of the GO ink, Ni─Co precursor ink and KMnO_4_ ink on bamboo fabric substrate e) CV curves of MnO_2_─NiCo_2_O_4_//rGO asymmetric device at different scan rates f) GCD profiles of the MnO_2_─NiCo_2_O_4_//rGO asymmetric device at various current densities and g) CV curves of the device under different mechanical bending conditions (inset image shows digital photograph of textile supercapacitor. Reproduced with permission.^[^ ^204^ ^]^ Copyright 2020, The Author(s).

In addition to the rapid development of high‐performance active materials for supercapacitor electrode fabrication, attention should also be paid to the eco‐friendliness and sustainability of the fabrication processes. The conventional fabrication processes rely on several toxic chemicals or solvents, involving considerable waste of precious functional materials, and/or release of toxic by‐products. Inkjet printing, in this regard, is considered as a sustainable technique due to its combination of multiple merits, such as purely additive processing, direct (mask‐free) patterning, high resolution, minimized material waste, good scalability, and excellent compatibility with versatility in active materials and substrates selection.^[^ ^448^ ^]^ Therefore, inkjet printing has been in increased focus of the e‐textile manufacturers, specially for supercapacitors on several substrates such as PET,^[^ ^449^, ^450^, ^451^, ^452^, ^453^, ^454^, ^455^ ^]^ flexible ITO,^[^ ^450^ ^]^ PI,^[^ ^456^, ^457^ ^]^ Kapton,^[^ ^453^, ^454^, ^458^ ^]^ glass,^[^ ^454^ ^]^ paper,^[^ ^459^ ^]^ PDMS etc. Giannakou et al.,^[^ ^447^ ^]^ deposited NiO coplanar electrodes on flexible substrate through the inkjet printing method. The silver nanoparticle ink was inkjet‐printed first on a flexible PET substrate in a coplanar interdigitated configuration to form the current collector of the device. Then the NiO nanoparticle ink was printed on top of the interdigitated silver current collector. The structure was annealed to promote nanoparticle sintering of both layers, the electrolyte was drop‐cast on top of the active interdigitated region of the device, **Figure** **17**a–c. The highest areal capacitance was reported by Sundriyal et al.,^[^ ^204^ ^]^ who demonstrated the inkjet‐printing of the rGO and metal precursors of MnO_2_ and NiCO_2_O_4_ over the bamboo fabric substrates. Developed MnO_2_–NiCo_2_O_4_//rGO printed asymmetric supercapacitors exhibited a high areal capacitance of 2.12 Fcm^−2^, excellent energy density of 37.8 mW cm^−3,^ and power density of 2678.4 mW cm^−3^, good cycle life and high retention. It also indicated no structural failure and capacitance loss under different mechanical deformation conditions, Figure 17d–g. Zhang et al.^[^ ^454^ ^]^ reported the highest volumetric capacitance of 562 Fcm^−3^ for inkjet‐printed supercapacitors. They demonstrated two types of 2D titanium carbide (Ti_3_C_2_T_x_) MXene inks, in the absence of any additive or binary‐solvent systems high resolution and spatially uniform inkjet printing on AlO x‐coated PET, glass, Kapton substrates.

### 5.4 Printed E‐Textiles for Personalized Thermoregulation

Thermal comfort is a condition of mind, expressing satisfaction with the thermal environment.^[^ ^460^, ^461^, ^462^, ^463^, ^464^, ^465^ ^]^ It represents whether a person feels neither too cold nor too warm. Thermal conditions of the human body are crucial for both physical and psychological health and may become potentially life‐threatening if the core body temperature reaches conditions of hyperthermia (above 37.5 °C−38.3 °C) or hypothermia (below 35 °C).^[^ ^466^ ^]^ Maintaining human body thermal comfort wisely is important for efficient human body energy management, therefore, wearable heaters have recently attracted great interest with the growing demand for wearable and stretchable devices.^[^ ^467^ ^]^ The introduction of heating systems made directly from textiles could potentially help humans maintain comfort without using any additional bulky heating arrangement. The main advantage of this improvement is the development of heated materials with built‐in flexibility provided by textile structures. With the progressive development of conductive threads, textile‐heating systems represent one of the major growing sectors in textiles.^[^ ^468^ ^]^ Such heating elements are used in climate control and personal thermal management,^[^ ^469^ ^]^ such as protecting us against cold stress both in foul weather outdoor environments and in indoor environments by raising the human body's temperature applied to normal clothes.^[^ ^470^ ^]^ A personal heating garment (PHG) is helpful to reduce cold stress and freezing (e.g., frostbite and frostnip) or non‐freezing cold injuries (e.g., immersion foot, cracked skin and chilblains caused by chilling of extremities, usually fingers, toes, and ears, etc.).^[^ ^471^ ^]^ Heating of smart clothing products based on nontextile electronic components already exists in the market, but these products do not have a big consumer demand yet.^[^ ^472^ ^]^ Heating elements can have applications including heated garments, heated molds for industrial use, heated furniture, electrically heated cushions and heated blankets, heating elements for the automotive sector, heating solutions for medicinal purposes, surface heating elements in general, etc. For wearable applications, conducting panels can be used as heating elements. The heating rate is given by the power P that is generated due to Joule heating with current I, running through the conductor with resistance *R*.^[^ ^136^ ^]^

(10) $$P=RI^{2}$$

Several methods have been reported for the fabrication of textile heaters, such as chemical deposition of silver nanoparticles (AgNPs) on widely used cotton fabric,^[^ ^150^ ^]^ wrapping of AgNW on yarn,^[^ ^473^ ^]^ weaving,^[^ ^474^ ^]^ braiding^[^ ^475^ ^]^ or knitting^[^ ^476^ ^]^ of conductive yarns, etc. However, the coating technique was extensively studied for realizing heating textiles. Textiles coated with several carbonaceous materials,^[^ ^477^, ^478^, ^479^ ^]^ conducting polymers,^[^ ^480^, ^481^, ^482^ ^]^ metals,^[^ ^483^, ^484^, ^485^ ^]^ MXenes,^[^ ^486^, ^487^ ^]^ etc were reported by several research groups. **Table** **13** summarizes the textile‐based printed heaters.

**Table 13:** Summary of printed e‐textiles for personalized thermoregulation.

Printing method	Substrate (Reporting year)	Composed of	Maximum Temperature	Performance	Reference
Screen print	Polyester‐cotton (2012)	Ag as conductor layer and encapsulation material	120 °C using a 30V input voltage, 35–40 °C using a 12 V battery		[144]
	PET (2019)	PEDOT:PSS, AgNW	99 °C at 12 V and 107°C at 54 V within 20 s		[488]
	Stretchable textile (2021)	Positive temperature coefficient (PTC) powder and MWCNTs with embroidered electrodes	56.1 °C with a power consumption of 5 W over 7 min	Heat generation maintained 95% after 100 000 cycles of 20% stretch–contraction testing. Heating temperature remained uniformly distributed within ± 2 °C	[489]
Screen and transfer printing	Polystyrene–block–polyisoprene–block–polystyrene (SIS)	Silver fractal dendrites (Ag FDs) conductive ink	52.3 °C at 1 V	Low‐voltage driving Joule heating performance	[490]
Electrohydrodynamic printing	Polyethylene terephthalate (PET), paper, glass, polydimethylsiloxane (PDMS) (2018)	AgNW ink	≈160 °C at the voltage of 25 V	Maximum heating rate of ≈21 °C S^−1^ and cooling rate of ≈29 °C S^−1^.	[491]
Patternable spray coating	Stretchable fabric (2019)	Ag nanowire/carbon nanotube composites	35 °C‐55 °C at 3–5 V		[492]
Scalpel printing	Cotton, polyester woven and nonwoven (2019)	Nano carbon colloidal ink of MWCNT synthesized by globular protein serum bovine albumin (BSA)	Organic NC printed cotton woven fabrics 140 °C at 20 V		[493]
Pattern printing	Stretchable LM@PDMS (2019)	conductive composite of liquid‐metal (LM) and polydimethylsiloxane (PDMS)	45.26 and 95.9 °C, at 2.0 and 3.5 V	Suitable as a stretchable wearable electrically driven heater (WEDH) for wearable thermotherapy	[494]

**Figure 18:**
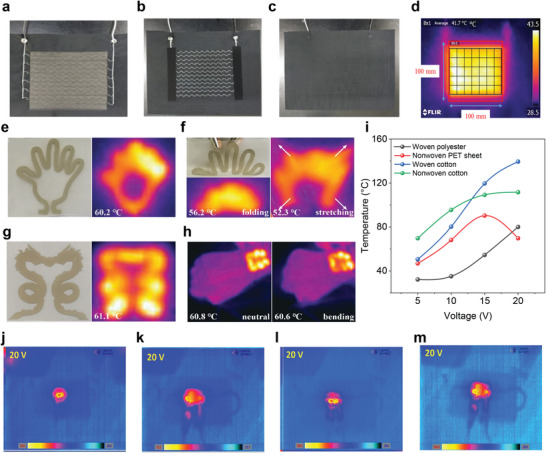
Printed E‐textiles for personalized Thermoregulation. Digital photographs of the a) Front b) Back after screen‐printing; and c) The finished product. (d) Heating temperature uniformity across the PTC‐SHT at an applied voltage of 3 V. Reproduced with permission.^[^ ^489^ ^]^ Copyright 2021, The authors. e) The digital photograph and IR image of the multifunctional wearable electronics with palm pattern, and f) Its IR images under folding and stretching at 3 V. g) The digital photograph and IR image of the MWE with double‐dragon pattern (top), and h) Its IR images of the pattern attached to the wrist in a natural and bent state (bottom) at 2 V. Reproduced with permission.^[^ ^490^ ^]^ Copyright 2019, WILEY‐VCH. i) Voltage versus surface Temperature diagram of different types of Organic nanocarbon ink (NC) printed fabrics. Measurement of surface temperature using thermal expert camera for j) Cotton woven k) Polyester woven l) Cotton nonwoven and m) PET sheet at 20V. Reproduced with permission.^[^ ^493^ ^]^ Copyright 2018, Elsevier Inc.

Torah et al.,^[^ ^144^ ^]^ reported for the first time a flexible heater screen‐printed directly onto polyester‐cotton fabric. They printed an initial insulation layer to reduce the roughness of the textile thus improving the surface quality and electrically insulating the subsequently printed heating conductor. A conductive layer was then printed onto the insulation layer to provide the heating element. Finally, another insulation layer was printed on top to provide complete electrical insulation of the conductor layer. The fabric was successfully heated up to 120 °C using a 30V input voltage. Choi et al.,^[^ ^489^ ^]^ in 2021 fabricated a high‐stretch PTC surface heating textile (PTC‐SHT) by screen‐printing using a composite paste of PTC powder and multiwall carbon nanotubes (MWCNTs) onto a high‐stretch textile with embroidered electrodes, **Figure** **18**a–d. Overall, the temperature increased to 56.1 °C with a power consumption of 5 W over 7 min. The heat generation characteristics were maintained at 95% after 100 000 cycles of 20% stretch–contraction testing, and the heating temperature remained uniformly distributed within ±2 °C across the entire heating element.

Tian et al.,^[^ ^490^ ^]^ utilized both screen and transfer printing for silver fractal dendrites (Ag FDs) conductive ink on the polystyrene–block–polyisoprene–block–polystyrene (SIS) thin film. The ultra‐stretchable flexible printed pattern was demonstrated as a low‐voltage driving Joule heating performance (heated to 52.3 °C at 1 V). They also reported their heater applications in the bent state, Figure 18e–h. Cui et al.,^[^ ^491^ ^]^ reported an electrohydrodynamic printing of Ag‐NW ink on several flexible substrates including PET, paper, glass, and PDMS. The printed heater obtained a temperature of up to ≈160 °C at the voltage of 25 V with a maximum heating rate of ≈21 °C s^−1^ and a cooling rate of ≈29 °C s^−1^. Arbab et al.,^[^ ^493^ ^]^ reported the development of a printable carbon ink of multiwall carbon nanotubes (MWCNT) synthesized by globular protein serum bovine albumin (BSA). The maximum rise in temperature of cotton and polyester, woven, and nonwoven fabrics was reported at 140  °C at 20 V, Figure 18i–m. Though not on textiles, Choi et al.^[^ ^495^ ^]^ proposed a transfer printing technique to integrate graphene‐based stretchable sensors, actuators, light‐emitting diodes, and other electronics in one platform, paving the way toward transparent and wearable multifunctional electronic systems.

### 5.5 Printed Textile Electrochromics

Electrochromic materials, also called switchable materials, change color in a persistent but reversible manner by an electrochemical reaction, and the phenomenon is called electrochromism. The visible change in transmittance and/or reflectance is associated with an electrochemically induced oxidation‐reduction reaction. It results from the generation of different visible region electronic absorption bands on switching between redox states.^[^ ^496^ ^]^ They are usually used in the form of thin film devices such as in electrochromic, or smart windows, architectural glazing, automotive mirrors, rearview mirrors, sunroofs, sunglasses, and other high‐end applications.^[^ ^497^ ^]^ Electrochromic materials usually include viologens, transition metal oxides (WO_3_, MoO_3_, V_2_O_5_, Nb_2_O_5_), metal hexacyanometallates, conducting polymers, etc. Such materials can also be introduced in textiles to develop color‐changing and light‐emitting textiles. Upon application of specific stimuli, such textiles can change their optical properties. Some of these textile‐based structures have already been used for the development of flexible displays, which can be used for multiple applications, from medical textiles to communicative textiles, as well as for art and fashion.^[^ ^498^ ^]^

**Figure 19:**
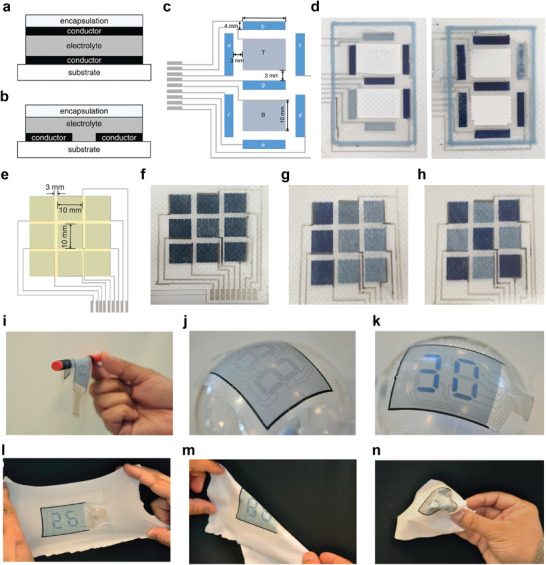
Printed textile electrochromic devices, a) Vertical b) Lateral arrangement. c) Cross‐section of seven segment EC display d) Photographs of numbers displayed on EC display‐ Four and Six, e) Design layout and f) Printed sample of 3 × 3 matrix display g and h) Examples of EC display with pixels activated Each pixel was able to change between pale blue and dark blue ≈5 s. Reproduced with permission.^[^ ^502^ ^]^ Copyright 2020, The Institution of Engineering and Technology. Double 7‐segment display capable of showing any two‐digit number, i) The display has a thin, light, flexible form factor j) A display conforming to a round surface, due to its stretchability, is shown in the off‐state, and k) In the on‐state as the number ‘30′ l) Functional display attached to elastic textile following the deformation of the fabric upon stretching m) Folding and n) Crumpling. Reproduced with permission.^[^ ^503^ ^]^ Copyright 2021, The Author(s).

Researchers fabricated electrochromic textiles by coating PEDOT:PSS on readymade polyester flex printing fabric,^[^ ^499^ ^]^ electrochemical deposition of PANI on metal‐plated textiles,^[^ ^500^ ^]^ or spray coating of PEDOT:PSS on polyethylene polyethylene terephthalate (PEPES) membranes.^[^ ^501^ ^]^ Wei et al.^[^ ^502^ ^]^ reported the design, fabrication, and testing of a dispenser‐printed electrochromic (EC) display on fabric using PEDOT:PSS. The display was directly printed onto a polyvinyl chloride (PVC)‐coated, 100% polyester woven fabric. Each display pixel, consisting of the color‐changing and counter electrodes, was separately driven at two voltage direct currents (VDCs). The color change between pale blue and dark blue was controlled by switching the polarity using a microcontroller. Two demonstrators, a 3 × 3‐pixel matrix display and a seven‐segment display, were achieved with an average switching speed of 5 s, **Figure** **19**a–h. Linderhed et al.^[^ ^503^ ^]^ presented a scalable screen printing process to produce stretchable electrochromic displays. Electrochromic PEDOT:PSS inks were screen printed on thermoplastic polyurethane substrate for the manufacturing of stretchable organic electronic devices that retained color contrast with useful switching times at static strains up to 50% and strain cycling up to 30% strain. A double‐digit 7‐segment ECDs were also produced, which could conform to curved surfaces and be mounted onto stretchable fabrics while remaining fully functional, Figure 19i–n.

### 5.6 Printed Textile Thermoelectrics

Thermoelectric (TE) generators are an excellent candidate for powering wearable electronics and the “Internet of Things,” due to their capability of directly converting heat to electrical energy.^[^ ^504^, ^505^ ^]^ Flexible TEs, can directly convert the heat from the human body into useful electricity, providing a promising solution for uninterrupted power to wearables.^[^ ^506^ ^]^ Sun et al.^[^ ^507^ ^]^ wrapped alternately doped CNT fibers with acrylic fibers and woven into π‐type thermoelectric modules. Printing techniques offer a scalable approach to fabricating TE devices on flexible substrates, especially textiles for power generation. For example, Kim et al.^[^ ^508^ ^]^ prepared p‐type Sb_2_Te_3_TE thick films and n‐type Bi_2_Te_3_ TE thick films on glass fiber fabric by screen printing. The thermoelectric conversion factor and ZT values of printed films were≈0.3 at room temperature. Copper foils were printed to interconnect the p‐type and n‐type TE thick films via a Ni peel‐off method to construct the TE generator device. Shin et al.,^[^ ^509^ ^]^ also reported a scalable screen printing of thermoelectric layers on flexible glass fiber fabrics. With high electrical conductivity and low thermal conductivity, the screen‐printed TE layers showed high room‐temperature *ZT* values of 0.65 and 0.81 for p‐type and n‐type, respectively. Assembling thermoelectric modules into the fabric to harvest energy from body heat could one‐day power multitudinous wearable electronics. However, the bottleneck of the printed TE devices still lies in the low performance of the printed TE materials.^[^ ^509^ ^]^

### 5.7 Printed Textile Optoelectronics

Optoelectronics concerns the study and application of electronic devices that source, detect, and control light. Optoelectronic devices consist of different semiconductor alloys lying on substrates. During the growth of the multi‐quantum well of a laser‐active region, different layers of semiconductors are sequentially deposited onto the substrate, alternating between well and barrier regions. In well regions, electrons and holes are recombined to provide the laser light, while barrier regions are important for confining the electrons and holes inside the wells.^[^ ^510^ ^]^ Incorporating such optoelectronic devices into textiles can increase fabric capabilities and functions, such as fabric‐based communications or physiological monitoring.^[^ ^511^ ^]^ Most of the reported textile‐based optoelectronics are based on embedding multi‐material fibers into textiles. The ability to integrate complex electronic and optoelectronic functionalities within soft and thin fibers is one of today's key advanced manufacturing challenges. Multifunctional and connected fiber devices thus will be at the heart of the development of smart textiles and wearable devices.^[^ ^512^ ^]^ High‐speed fiber LED transmitters and photodetectors present an opportunity for high‐bandwidth inter‐fiber communication links. Rein et al.,^[^ ^511^ ^]^ demonstrated a scalable thermal drawing process of electrically connected diode fibers to construct a macroscopic preform that hosts discrete diodes. Conducting copper or tungsten wires were fed into its inner structure. As the preform is heated and drawn into a fiber, the conducting wires approach the diodes until they make electrical contact, resulting in hundreds of diodes connected in parallel inside a single fiber. They realized two types of in‐fiber devices; light‐emitting and photodetecting p–i–n diodes. They further embedded these fibers into a fabric. es. Finally, heart‐rate measurements with the diodes indicated their potential for implementation in all‐fabric physiological‐status monitoring systems. However, to the best of our knowledge, printing technology is not yet employed to realize any textile optoelectronics.

## 6 Conclusion and Perspective

**Figure 20:**
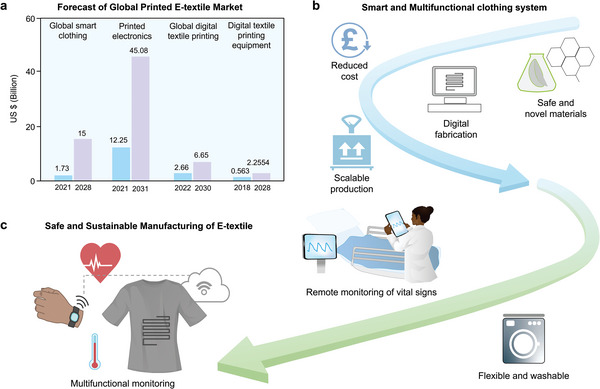
Future prospects and research direction of printed e‐textiles. a. Forecast of global printed e‐textiles market b. Smart and multifunctional clothing system and c. Safe and sustainable manufacturing of e‐textiles.

The rapid advancement of the Internet of Things has put people in need of intelligent and controllable multifunctional electronic devices to be utilized in fulfilling different requirements in real life. We expect that textile‐based printed flexible and wearable electronics will lead to a revolution in energy and healthcare. In the recent past, they have already gained huge attention due to their softness, breathability, biocompatibility, and durability.^[^ ^513^, ^514^ ^]^ Acumen Research and Consulting says the global e‐textiles and smart clothing market size is estimated to grow a CAGR above 32.3% over time and reach a market value of ≈US$15 Bn by 2028.^[^ ^515^ ^]^ On the other hand, Printed electronics market was estimated by Transparency Market Research, they reported the global printed electronics market was valued at US$ 12.25 Bn in 2021 and is estimated to advance at a CAGR of 14.2% from 2022 to 2031 to an expected reach of US$ 45.08 Bn by the end of 2031.^[^ ^516^ ^]^ Research and Market estimate the global digital textile printing market of ≈US$ 2.66 Billion in 2022, with a forecast to grow at a CAGR of 12.1% reaching up to US$ 6.65 Billion in 2030.^[^ ^517^ ^]^ Persistent Market Research expects to attain a value of US$ 2255.4 Mn for the global market of digital textile printing equipment with growth of a CAGR of 14.9% between 2018 and 2028,^[^ ^518^ ^]^ **Figure** **20**a.

There are several considerations that need to be addressed before the widescale commercial adoption of wearable electronic textiles. Most of the existing wearable e‐textiles can perform only a single functionality, i.e., either as an ECG or temperature or any other sensor; however, a textile that can monitor several health parameters is of special interest for personalized healthcare applications,^[^ ^519^, ^520^ ^]^ Figure 20b. Future research in textile wearable electronics will be directed to the integration of energy generation, storing and powering the sensors, actuators, electrochromic, shape memory, and even self‐repair functionality within the same clothing.^[^ ^521^ ^]^ Integration of several electronic components in a multifunctional e‐textile is also considered challenging. However, the traditional cut‐and‐sew method is the simplest method to integrate all the fabric‐based electronic devices into final textile products. Adhesive bonding, ultrasonic welding, and laser welding are other joining methods able to eliminate bulky stitched seams and bring less damage to the electronic components within the devices.^[^ ^522^ ^]^

Another consideration of wearable e‐textiles is flexibility. The textile substrate is naturally flexible, however, the flexibility of the electrode material to construct the electronic components is the prime concern. Enhancing the electrical performance often requires the deposition of additional conductive material which results in a more rigid platform. Therefore, further exploration in materials or design aspects is still needed, which could possess ultra‐flexibility while still keeping ultra‐high electrical performances. Washability is often seen as one of the main obstacles to reaching a wider market of e‐textile products. Most of the experimental designs lack this criterion hindering the scope of any lab‐based device to be used commercially, Figure 20b. To assess, improve, and evaluate the extent of e‐textiles in terms of washing, repeated test cycles are executed. So far, there are no standardized methods for testing the wash fastness of e‐textiles and no protocols to comparably assess the washability of tested products.^[^ ^523^ ^]^ Washing e‐textiles is challenging; the effect of washing on the performance relies not only on the type of conductive materials or fabrication process but also on the specific textile substrate used (i.e., materials and constructions) along with their interdependency. As such, no global conclusion can be drawn on how a washing program for smart e‐textiles should be configured. Considering textile substrates, the applicability and suitability of different textiles depend on the type of conductive track used while looking to achieve the best washability results. On the other hand, if the choice of textile for a smart textile application is fixed due to specific requirements (such as sufficient elasticity for sports clothing, etc.), the type of conductive track used needs to be adapted accordingly for best reliable results.^[^ ^149^ ^]^ A fully integrated multifunctional clothing system might be connected with a cloud system which would facilitate the remote monitoring of health parameters for any patient, elderly, or childcare, Figure 20b.

The most important criterion for the fabrication of textile‐based wearable electronics is safety. The primary utilization of such devices is aimed at human health management; therefore, the final device must be safe for the human body as well as for the environment during disposal. Concerns should always be prioritized to find safer and environmentally friendly substitutes than the existing toxic, environmentally unfriendly, and non‐bio‐compatible materials, Figure 20c.

With the increased attention toward the use of more and more clean energy, the consumer culture around the world has also raised the need for all products to be more sustainable and recyclable to reduce the environmental impact.^[^ ^524^ ^]^ This is valid for the wearable electronics industry too.^[^ ^525^, ^526^ ^]^ Therefore, the need to explore safe and sustainable manufacturing of electronic devices is an imperative concern of the world today. New eco‐friendly, as well as cost‐efficient electronic systems, have to be developed, in view of the requirements of emerging ecological concerns and modern society.^[^ ^527^ ^]^ Digital fabrication, i.e., inkjet printing is a promising sustainable solution to reduce the material waste and environmental effects of the conventional printing systems, Figure 20c. There are still some limitations in different aspects of inkjet printing including printing speed, cost–benefit issues, printed film uniformity, and fluids' jet‐ability as ink—overcoming which would be the future prospect of this leading technology.^[^ ^190^ ^]^

Another very important consideration for the commercial adoption of the wearable electronics industry is the improvement of performance along with the reduction of production costs. For the fabrication of electronic components, replacing an existing material with a new low‐cost raw material, such as natural mineral resources could be an attractive option. The combination of low‐price raw materials with high‐price raw materials without compromising the performance could be another approach to reduce the overall cost,^[^ ^137^ ^]^ Figure 20c.

The textile electrodes to be integrated into the full garment must be scalable for industrial manufacturing. Most of the existing reports on wearable e‐textiles are laboratory‐based, and addressing all these issues might direct the e‐textile industry into large‐scale adaptation of e‐textiles, Figure 20c. We assume the development of wearable e‐textiles will be more rapid and far‐reaching with the popularity of new and smart wearable devices.^[^ ^528^ ^]^

## Conflict of Interest

The authors declare no conflict of interest.
